# Linking acetylated α-Tubulin redistribution to α-Synuclein pathology in brain of Parkinson’s disease patients

**DOI:** 10.1038/s41531-023-00607-9

**Published:** 2024-01-02

**Authors:** Samanta Mazzetti, Federica Giampietro, Alessandra Maria Calogero, Huseyin Berkcan Isilgan, Gloria Gagliardi, Chiara Rolando, Francesca Cantele, Miriam Ascagni, Manuela Bramerio, Giorgio Giaccone, Ioannis Ugo Isaias, Gianni Pezzoli, Graziella Cappelletti

**Affiliations:** 1 Department of Biosciences, Università degli Studi di Milano, Milan, Italy; 2 Fondazione Grigioni per il Morbo di Parkinson, Milan, Italy; 3 Department of Chemistry, Università degli Studi di Milano, Milan, Italy; 4 Unitech NOLIMITS, Università degli Studi di Milano, Milan, Italy; 5 S. C. Divisione Oncologia Falck and S. C. Divisione Anatomia Patologica, Ospedale Niguarda Ca’ Granda, Milan, Italy; 6 Unit of Neuropathology and Neurology, Fondazione IRCCS Istituto Neurologico Carlo Besta, Milan, Italy; 7 Parkinson Institute, ASST G. Pini-CTO, Milan, Milan, Italy; 8 Department of Neurology, University Hospital of Würzburg and the Julius Maximilian University of Würzburg, 97080 Würzburg, Germany; 9 Center of Excellence on Neurodegenerative Diseases, Università degli Studi di Milano, Milan, Italy

**Keywords:** Parkinson's disease, Parkinson's disease, Brain, Neurodegeneration

## Abstract

Highly specialized microtubules in neurons are crucial to both health and disease of the nervous system, and their properties are strictly regulated by different post-translational modifications, including α-Tubulin acetylation. An imbalance in the levels of acetylated α-Tubulin has been reported in experimental models of Parkinson’s disease (PD) whereas pharmacological or genetic modulation that leads to increased acetylated α-Tubulin successfully rescues axonal transport defects and inhibits α-Synuclein aggregation. However, the role of acetylation of α-Tubulin in the human nervous system is largely unknown as most studies are based on in vitro evidence. To capture the complexity of the pathological processes in vivo, we analysed *post-mortem* human brain of PD patients and control subjects. In the brain of PD patients at Braak stage 6, we found a redistribution of acetylated α-Tubulin, which accumulates in the neuronal cell bodies in subcortical structures but not in the cerebral cortex, and decreases in the axonal compartment, both in putamen bundles of fibres and in sudomotor fibres. High-resolution and 3D reconstruction analysis linked acetylated α-Tubulin redistribution to α-Synuclein oligomerization and to phosphorylated Ser 129 α-Synuclein, leading us to propose a model for Lewy body (LB) formation. Finally, in *post-mortem* human brain, we observed threadlike structures, resembling tunnelling nanotubes that contain α-Synuclein oligomers and are associated with acetylated α-Tubulin enriched neurons. In conclusion, we support the role of acetylated α-Tubulin in PD pathogenesis and LB formation.

## Introduction

Cytoskeleton is a pivotal structural component in neurons, where it regulates the architecture and also orchestrates many intracellular events^1^. Neuronal microtubules are cytoskeletal filaments that are essential for the health of the nervous system not only during neurodevelopment but also in mature neurons as they enable a high degree of morphological and functional complexity, regulate the trafficking of molecules and organelles, and control synaptic plasticity and dendritic spine structure^2–4^. In the last two decades, microtubule imbalance has emerged as a player in diseases of the nervous system. Different mutations in tubulin genes are linked to a set of neurological disorders, mainly featured by cortical malformation^5^. Moreover, loss of microtubules or changes in their stability and dynamics may contribute to neurodegeneration as happens in Amyotrophic Lateral Sclerosis, Alzheimer’s disease and Parkinson’s disease (PD)^6–10^.

Neuronal microtubules are finely regulated by the “tubulin code” that is the result of different α- and β- tubulin isoforms and a plethora of post-translation modifications (PTMs), which controls both the structure and specific functions of each microtubule^11–13^. Among these PTMs, acetylation of tubulin has been widely investigated. Lysine 40 acetylation of α-tubulin, found in *Chlamydomonas flagella*^14^, is fundamental for neuronal architecture during development^4,15,16^, and is largely present in the axon initial segment and in the axon in mature neurons^17^. Recently, it has been demonstrated that acetylated α-tubulin enhances microtubule flexibility making them more resilient, protecting them against mechanical aging and stresses^18,19^. In addition, many cellular processes rely on acetylated α-tubulin, including cell migration^20^, autophagy^21^, intracellular trafficking^22^ and cell adhesion^23^.

The defective regulation of acetylated α-tubulin has been linked to PD^24–26^. Studies with 1-methyl-4-phenyl-1,2,3,6-tetrahydropyridine (MPTP), and its toxic metabolite 1-methyl-4-phenylpiridinium (MPP^+^) have shown that the imbalance of acetylated α-tubulin drives cell degeneration. In fact, MPP^+^ increases the fraction of acetylated α-tubulin and induces defects in axonal transport and mitochondrial damage in PC12 cells^27^ and in murine primary dopaminergic cultures^28^. Furthermore, mice treated with MPTP showed an early imbalance of acetylated α-tubulin in nigrostriatal dopaminergic fibres. This is rescued by Epothilone D, a microtubule stabilizer, which attenuates nigrostriatal degeneration showing that microtubule stabilization is neuroprotective^29^. In addition, recent biochemical data have shown a decrease in acetylated α-tubulin in the *substantia nigra* and no alteration in the cerebral cortex of *post-mortem* human brain obtained from PD patients compared to controls^30^.

PD is the most common neurodegenerative movement disorder^31,32^, and is characterized by a set of motor and non-motor symptoms and by α-Synuclein aggregation into Lewy bodies (LBs) and Lewy neurites^33–35^. α-Synuclein aggregation implies the formation of different pathological species including oligomers and fibrils^36^. α-Synuclein cell-to-cell spreading has been indicated as the route of disease propagation^37,38^. Studies in cellular models revealed that the transfer can occur thought tunnelling nanotubes, exosomes and endocytosis^39–41^. α-Synuclein aggregation has been linked to the impairment of different cellular processes including regulation of lysosomal and proteasomal functions, mitochondrial activity, biological membrane and synaptic activity, cytoskeleton assembly and functions^26,31,32^. Some data in human brain have pointed to α-Synuclein aggregation as a process characterized by different stages, starting with α-Synuclein that accumulates diffusely in neuronal cytoplasm. It then forms a shapeless and irregular aggregate with moderate α-Synuclein staining, and later acquires a well-defined round shape with discrete staining for α-Synuclein in the so-called pale body stage. Finally, it forms an aggregate with a central core and a ring-shaped surrounding halo positive for α-Synuclein that is a typical LB^42–44^. Interestingly, thanks also to advanced electron microscopy, LBs are defined as crowded aggregates, which in addition to proteins, also contain lipid membranes, vesicular structures, and dysmorphic organelles such as mitochondria and lysosomes^45^. Very recently, Moors and co-workers successfully used super-resolution microscopy to study the subcellular arrangement of α-Synuclein in PD human brain and demonstrated the presence of an onion skin-type LB containing a well-organized cytoskeletal framework associated with phosphorylated Ser129 (Ser129 P) α-Synuclein^46^. Neurofilament and β-Tubulin are distributed mainly in the peripheral part of this structure suggesting that such an organization is important in LB formation^46^. Additionally, Lewy neurites display cytoskeletal abnormalities due to the possible disruption in the neurofilament network^45^.

Increasing evidence suggests that α-Synuclein can interact with the microtubular cytoskeleton^47–49^. Initially, α-Synuclein was found to co-immunoprecipitate with α-tubulin from zebra finch and murine forebrain extracts^50^. Next, Alim and colleagues not only confirmed this interaction by demonstrating that α-tubulin co-precipitates with α-Synuclein in the cytoplasm of rat brains^51^, but also showed that α-Synuclein impacts on microtubule assembly and polymerization in vitro^52^. In recent years, many studies have further supported this interaction based on in vitro and in cultured cell assays^49,53^, and also on Proximity Ligation Assay (PLA) in mouse brains and in *post-mortem* human brains^54^. Nevertheless, this interplay seems to be altered in pathological conditions^48^. Mutated forms of α-Synuclein impact negatively on microtubule assembly in vitro and in neuronal cell systems^49,52,55^ and α-Synuclein overexpression is associated with disruption of the microtubule network causing impairment of axonal transport and neurite degeneration^56^. As regards human tissues, very few and incomplete data are available. It has been proven that α-Tubulin^51^ as well as HDAC6, the main α-Tubulin deacetylase, and its phosphorylated active form^57^ are localised in LBs. However, the mechanism that links α-Tubulin and α-Synuclein during the formation of aggregates warrants further investigation.

Our study shows that acetylated α-Tubulin is strongly redistributed in PD brains. First, we identified an accumulation of acetylated α-Tubulin in neuronal cell bodies and a decrease in axons both in the central and peripheral nervous system. We also correlated acetylated α-Tubulin redistribution with LB formation and analysed in depth the link with α-Synuclein oligomers and Ser129 P α-Synuclein. Interestingly, we highlighted a driving role of acetylated α-Tubulin during LB formation in PD human brain.

## Results

### Neuronal acetylated α-Tubulin rearrangement in the *substantia nigra* of PD patients

**Fig. 1:**
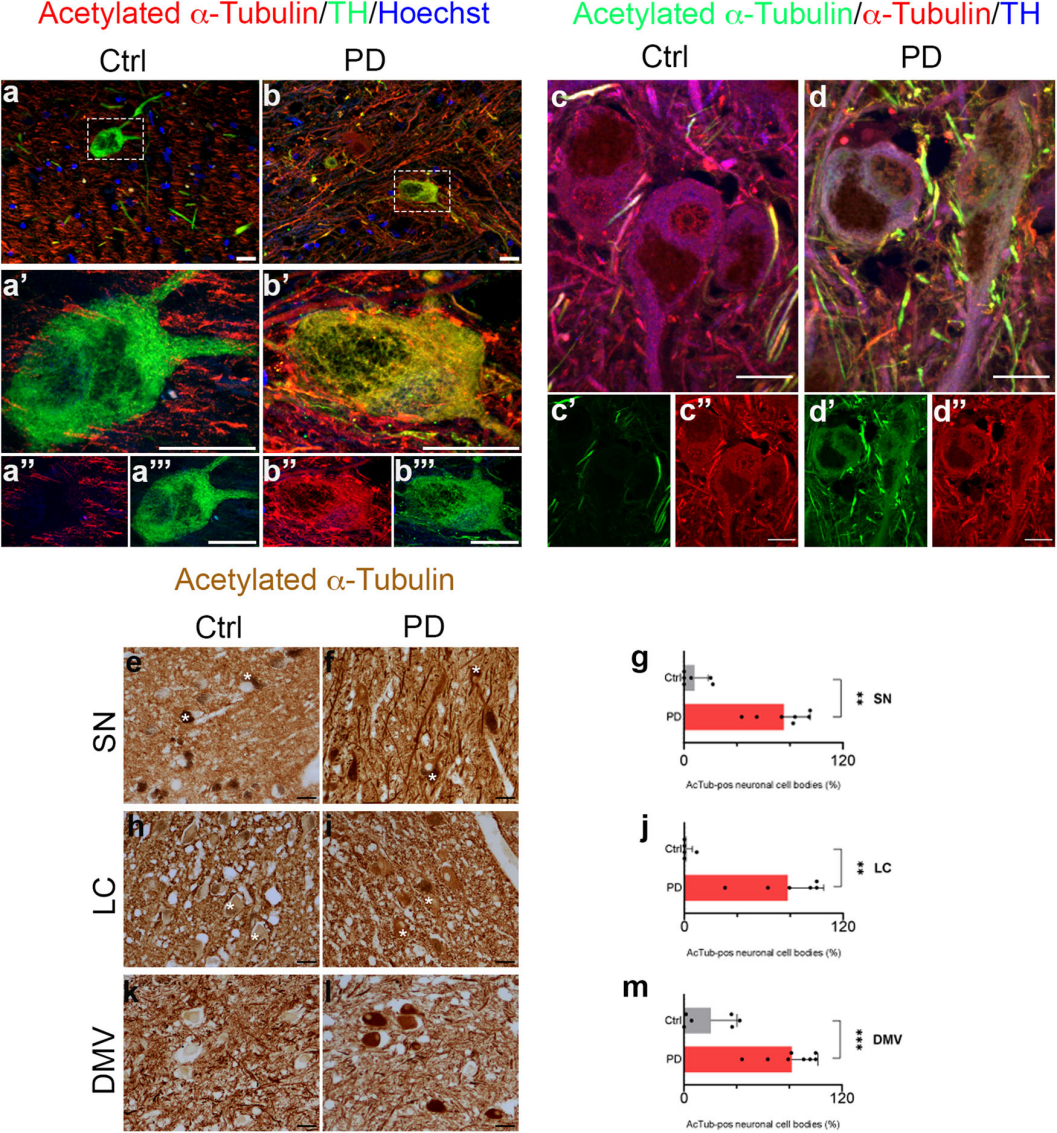
Acetylated α-Tubulin distribution in *post-mortem* human brain. Acetylated α-Tubulin distribution in dopaminergic neurons of *substantia nigra* in *post-mortem* human brain of control subjects (Ctrl) and PD patients (PD). Acetylated α-Tubulin (clone 6-11B-1) is localised only in neuropil of control samples (**a**–**a”’**) while it strongly accumulates within the cell body of dopaminergic neurons (TH positive) in PD (**b**–**b”’**). Nuclei are counterstained with Hoechst. Scale bar, 20 μm. **c**, **d”’** Total α-Tubulin staining is present in the cell bodies of both Ctrl and PD neurons, while the presence of acetylated α-Tubulin (clone D20G3) only in PD samples (**d**–**d”’**) is confirmed. Scale bar, 20 μm. **e**–**m** Control samples show acetylated α-Tubulin only in neuropil and in axonal fibres in SN (**e**) LC (**h**) and DMV (**k**). In the same regions of PD samples (**f**–**i**–**l**) acetylated α-Tubulin is strongly localised and accumulated in neuronal cytoplasm, and is also present in neuronal processes. White asterisks: neurons containing neuromelanin. Scale bar, 40 μm. The graphs show the percentage of acetylated α-Tubulin positive neuronal cell bodies ((**g**) SN Ctrl, *N* = 6, 263 neurons vs PD, *N* = 7, 496 neurons; (**j**) LC Ctrl, *N* = 6, 259 neurons vs PD, *N* = 6, 131 neurons; (**m**) DMV Ctrl, *N* = 6, 334 neurons vs PD, *N* = 8, 404 neurons). Data in graphs are reported as mean ± standard deviation. Mann-Whitney test, **p < 0.01; ***p < 0.001. SN: *substantia nigra*; LC: *locus coeruleus*; DMV: dorsal motor nucleus of vagus.

We analysed acetylated α-Tubulin distribution in the *substantia nigra*, which is the main region linked to the onset of motor symptoms in PD. In controls, we found that acetylated α-Tubulin is mainly present in cellular processes of neuropil, and surrounds tyrosine hydroxylase (TH) positive neurons, whose cell bodies are mostly negative (Fig. 1a–a”’). In contrast, in PD samples, acetylated α-Tubulin is enriched in the cellular body (Fig. 1b–b”’). Although we used the well characterized anti acetylated α-Tubulin antibody (clone 6-11B-1), we confirmed this pattern using a different acetylated α-Tubulin antibody (clone D20G3; Fig. 1c, c’, d, d’). Interestingly, the observed alterations in acetylated α-Tubulin do not match with changes in total α-Tubulin staining that is present in neuronal cell body of both control and PD patients (Fig. 1c”, d”).

Therefore, our first goal was to investigate whether this differential distribution of acetylated α-Tubulin is exclusive to neurons or could also be ascribed to glial cells, which are emerging players in PD^58^. We stained mesencephalic brain sections with acetylated α-Tubulin and glial markers: S100 Calcium Binding Protein β (S100β) for astrocytes, ionized calcium binding adaptor molecule 1 (IBA1) for microglia, and myelin basic protein (MBP) for oligodendrocytes (Supplementary Fig. 1). We observed that astrocytes display more ramifications in PD (Supplementary Fig. 1b, b”) than in controls (Supplementary Fig. 1a, a”). However, quantitative analysis did not reveal any significant difference between control subjects and PD patients in either the percentage of astrocytes positive for acetylated α-Tubulin (Supplementary Fig. 1c) or the co-localization between S100β and acetylated α-Tubulin (Mander’s coefficient; Supplementary Fig. 1d). As for microglia, which has been stated to be highly involved in the phagocytosis of damaged neurons^59^, the IBA1 marker is clearly visible in the cell bodies (Supplementary Fig. 1e, f”). Quantitative analysis revealed no significant difference between control subjects and PD patients in the percentage of microglial cell bodies positive for acetylated α-Tubulin (Supplementary Fig. 1g) and the co-localization between IBA1 and acetylated α-Tubulin (Mander’s coefficient; Supplementary Fig. 1h). Finally, using MBP, we found that acetylated α-Tubulin is mainly distributed along the neuropil in the *substantia nigra* in both control subjects (Supplementary Fig. 1 i, i”) and PD patients (Supplementary Fig. 1j, j”), while it is not present in oligodendrocytes. Quantitative analysis demonstrated no significant difference between control subjects and PD patients in terms of MBP co-localisation with acetylated α-Tubulin (Mander’s coefficient; Supplementary Fig. 1k). Collectively, these data reveal that in PD, localisation of acetylated α-Tubulin changes exclusively in neurons.

### Acetylated α-Tubulin mislocalizes in subcortical regions in PD patients

We analysed acetylated α-Tubulin in PD patients, starting from representative subcortical regions to the cerebral cortex^60^. In controls, acetylated α-Tubulin was mainly localised in the neuropil, as expected, and, rarely, in neuronal cellular bodies in the *substantia nigra* (Fig. 1e), *locus coeruleus* (Fig. 1h), and dorsal motor nucleus of vagus (Fig. 1k), while in PD, the percentage of neurons with a cell body strongly positive for acetylated α-Tubulin were abundant and greatly increased in all three regions (Fig. 1f, g, i, j, l, m). We confirmed the data in the subcortical regions, using a different acetylated α-Tubulin antibody (clone D20G3; Supplementary Fig. 2a–i), showing the significant increase in the percentage of acetylated α-Tubulin positive neurons in PD patients compared to controls (Supplementary Fig. 2c, f, i). Notably, this is not related to changes in the percentage of neuronal cell bodies positive for total α-Tubulin, that remains constant in both control and PD samples (Supplementary Fig. 2j–r).

On the other hand, no differences in acetylated α-Tubulin were observed in the cerebral cortex. Indeed, we investigated the frontal cortex (Supplementary Fig. 3a–e), cingulate cortex (Supplementary Fig. 3f–j) and entorhinal cortex (Supplementary Fig. 3k–o) and found that the staining was intense in the apical dendrites (Supplementary Fig. 3a, c, f, h, k, m) and in a limited number of pyramidal neurons (Supplementary Fig. 3b, d, g, i, l, n) in both control and PD samples. However, the non-significant changes in acetylated α-Tubulin (Supplementary Fig. 3e, j, o) could be due to the complexity of the cerebral cortex added to the intrinsic variability of human samples. We then investigated in-depth the compartmentalization of acetylated α-Tubulin in cortical neurons. First, we focused on apical dendrites. We double-stained frontal cortex slides with antibodies against acetylated α-Tubulin and MAP2, which is a marker of the somatodendritic compartment. MAP2 was present in the cellular bodies and apical dendrites both in control (Supplementary Fig. 3p, p”) and PD samples (Supplementary Fig. 3q, q”). Quantitative analysis revealed no significant difference between control subjects and PD patients in terms of either apical dendrites positive for acetylated α-Tubulin (Supplementary Fig. 3r) or co-localization between MAP2 and acetylated α-Tubulin (Mander’s coefficient; Supplementary Fig. 3s). Furthermore, we investigated the cortical white matter and found that no differences in the distribution of acetylated α-Tubulin are detectable among controls and PD patients (Supplementary Fig. 4).

Our data disclose the region-specific enrichment of acetylated α-Tubulin in neuronal cell body in PD patients.

### Acetylated α-Tubulin is reduced in fibre bundles of putamen

**Fig. 2:**
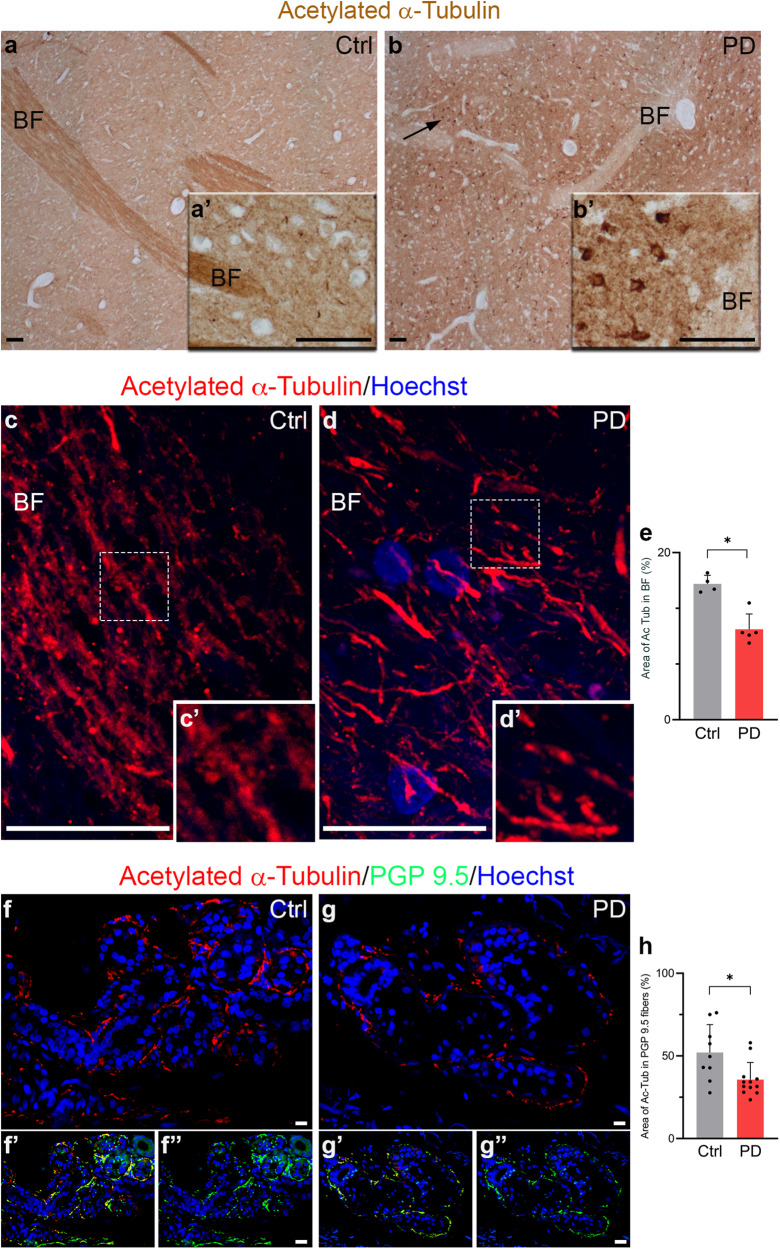
Acetylated α-Tubulin distribution in the fibres of the central and peripheral nervous system. **a**, **b** Acetylated α-Tubulin is localised in nigro-striatal fibres of putamen but not in neuronal cell bodies in controls (**a**–**a’**), whereas it is enriched mainly in neuronal cell bodies in PD patients (**b**, **b’**; black arrow). BF: bundle of fibres Scale bar, 100 μm. **c**–**e** In controls, acetylated α-Tubulin positive axons in putamen run parallel to each other (**c**, **c’**; dashed square: 2x magnified) while they are less dense in PD samples (**d**, **d’**; dashed square: 2x magnified). Nuclei are counterstained with Hoechst. The graphs show the percentage of the area covered by acetylated α-Tubulin (**e**; Ctrl, *N* = 3 vs PD, *N* = 4) in putamen BF. Scale bar, 20 μm. Data in graphs are reported as mean ± standard deviation. Mann-Whitney test, *p < 0.05. **f**–**h** Acetylated α-Tubulin localisation (**f**, **g**) within PGP 9.5 positive fibres (**f”**, **g”**) around sweat glands both in control subjects (**f**–**f”**) and PD patients (**g**–**g”**). Merge in (**f’**, **g’**). Nuclei are counterstained with Hoechst. The graph ((**h**) Ctrl, *N* = 9 vs PD, *N* = 12) indicates the percentage of the area covered by acetylated α-Tubulin in PGP 9.5 positive fibres. **f**, **g** Scale bar, 10 μm. **f’**, **f”**, **g’**, **g”**: Scale bar, 20 μm. Data in graphs are reported as mean ± standard deviation. Mann-Whitney test, *p < 0.05.

In addition to the cortex, we studied the bundle of fibres in the putamen, which plays a role in movement control through direct and indirect circuits. The axonal myelinated bundles, which cross the grey matter, displayed intense staining for acetylated α-Tubulin in control subjects (Fig. 2a), but were weakly stained in PD patients (Fig. 2b). On the contrary, neuron cell bodies lacked acetylated α-Tubulin in controls (Fig. 2a’) but were strongly positive in PD (Fig. 2b’).

We used confocal microscopy to perform quantitative analysis, evaluating acetylated α-Tubulin localisation in putamen bundles of fibres (Fig. 2c–d’). Acetylated α-Tubulin was significantly decreased in PD patients (Fig. 2e). This decrease in acetylated α-Tubulin in axons could be related to the increased number of neuronal cell bodies positive for acetylated α-Tubulin in the *substantia nigra*, that we observed and could suggest the translocation or redistribution of acetylated α-Tubulin from the axons to the cellular bodies.

### Acetylated α-Tubulin is reduced in PNS autonomic fibres in PD patients

Recently, PD has been defined as a multisystem disorder^61^ which does not only involve the central nervous system but also the peripheral nervous system. Notably, some studies have shown that α-Synuclein aggregation occurs in the autonomic structure of the skin in PD patients^62–64^. In particular, PD patients experienced sweating alterations, and we previously found a significant increase in α-Synuclein oligomers in the autonomic fibres innervating the sweat glands^64^. This led us to wonder whether the decrease in acetylated α-Tubulin that we saw in fibres of the central nervous system could be present also in the autonomic fibres that innervate the sweat glands in skin biopsies. We performed double immunofluorescence for acetylated α-Tubulin and PGP 9.5, which is a marker for fibres. We observed that acetylated α-Tubulin (Fig. 2f–f’, g–g’) localises inside PGP 9.5 positive fibres (Fig. 2f’-f”, g’-g”), which surround the sweat glands, both in control and PD samples. Quantitative analysis revealed a significant decrease in acetylated α-Tubulin in the fibres of PD patients (Fig. 2h) that is in line with results obtained in the putamen.

### Acetylated α-Tubulin interplay with α-Synuclein aggregation into LB

**Fig. 3:**
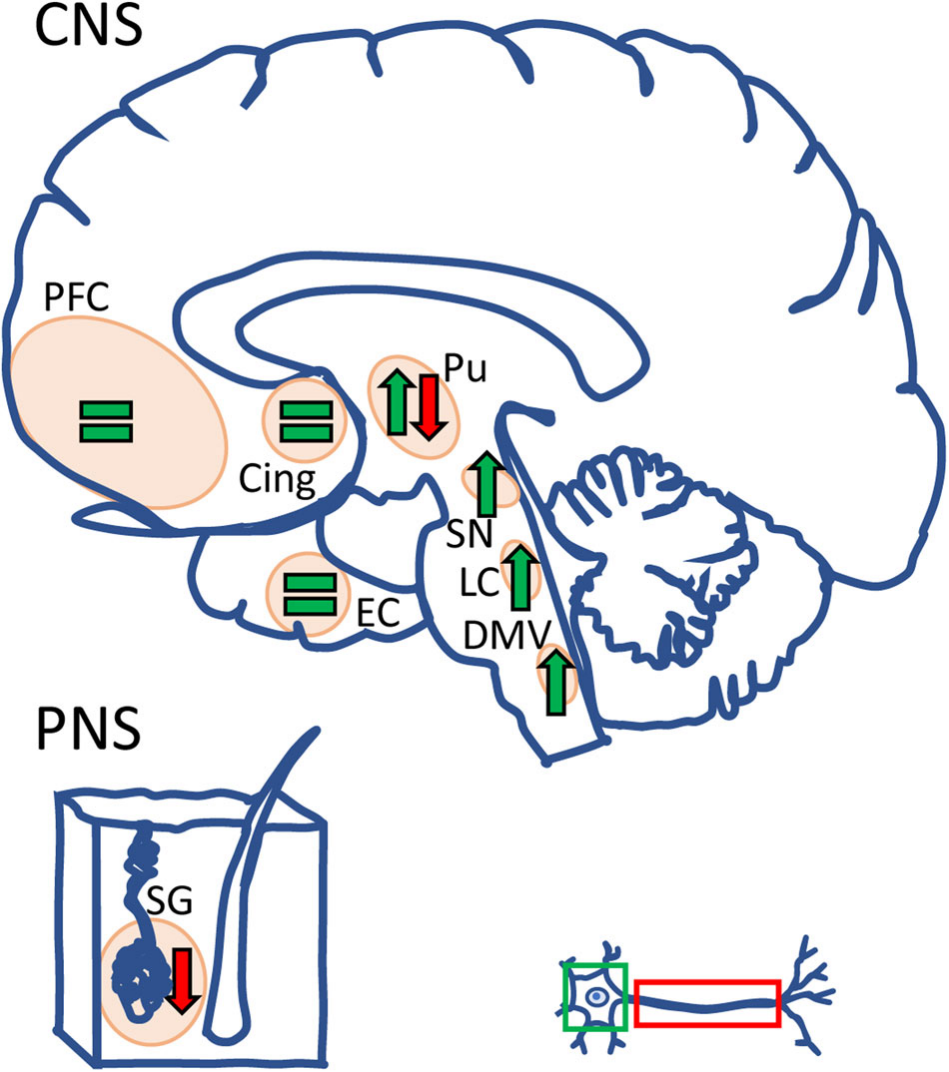
Schematic illustration of the neuroanatomical structures investigated in this study for acetylated α-Tubulin distribution. Both the central (CNS) and the peripheral nervous system (PNS) of PD patients were immunostained, analyzed, and the changes observed in the neuronal cell body and in the axonal compartment are indicated in green and red, respectively. Acetylated α-Tubulin is increased in the neuronal cell bodies in DMV, LC, SN and Putamen, whereas it remained unchanged in the cerebral cortex (PFC, Cing, and EC). Acetylated α-Tubulin is decreased in the axons of putamen and in the PNS namely in the axon innervating sweat gland. Cing cingulum; DMV dorsal motor nucleus of vagus; EC entorhinal cortex; LC locus coeruleus; SN substantia nigra; PFC prefrontal cortex; Pu putamen.

**Fig. 4:**
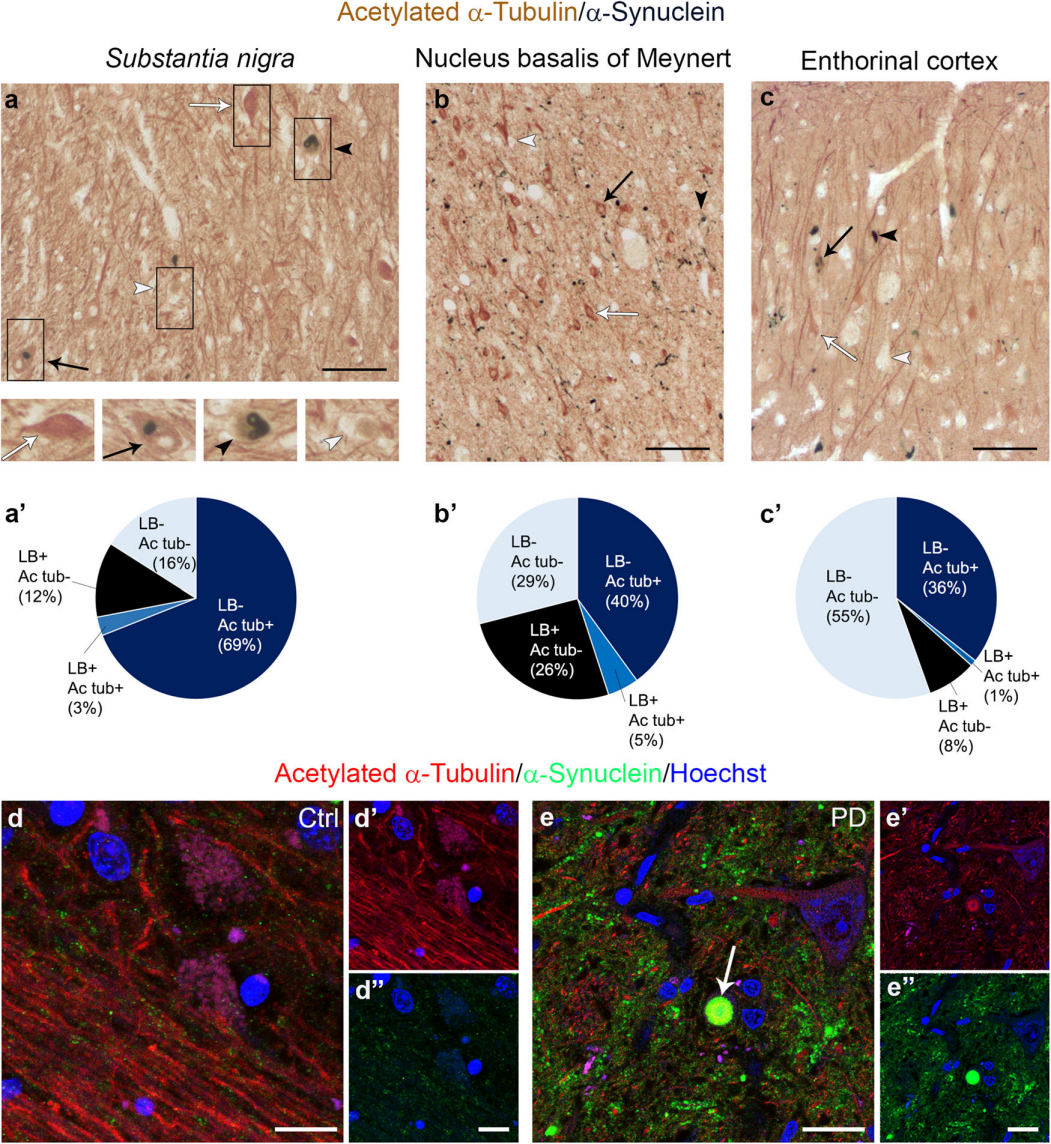
Linking acetylated α-Tubulin redistribution with α-Synuclein pathology in human brain. **a**–**c’** Four categories of neurons are distinguishable in the *substantia nigra* (**a** *N* = 3, 574 neurons), nucleus basalis of Meynert (**b** *N* = 3, 154 neurons) and entorhinal cortex (**c** *N* = 3, 1173 neurons) of PD patients: neurons exclusively positive for acetylated α-Tubulin (white arrow), neurons containing α-Synuclein accumulated as Lewy bodies (black arrowheads), neurons positive for both acetylated α-Tubulin and α-Synuclein (black arrow) and neurons lacking staining (asterisk). Scale bar, 100 μm. Insets show 1.5 x magnification of the representative neuronal cell bodies for the four categories. Pie charts (**a’**–**c’**) showing cell percentages for each category in the three regions. **d**, **e** In controls, acetylated α-Tubulin is present in fibres (**d**–**d’**) and α-Synuclein shows synaptic staining (**d**, **d”**). In PD samples, acetylated α-Tubulin accumulates in neuronal cell bodies and in few α-Synuclein positive aggregates (**e**–**e”**, white arrow). Nuclei are counterstained with Hoechst. Scale bar, 25 μm. Ac tub acetylated α-Tubulin; LB α-Synuclein positive-Lewy bodies.

Given the redistribution of acetylated α-Tubulin in PD (summarized in Fig. 3), we moved to investigate the relationship between α-Synuclein pathology and acetylated α-Tubulin using a double immunoenzymatic method in those regions where LBs are abundant^60^ and acetylated α-Tubulin accumulates in the soma: *substantia nigra* (Fig. 4a), nucleus basalis of Meynert (Fig. 4b), and entorhinal cortex (Fig. 4 c). In all these areas, four populations of neurons were identified and scored: (i) neurons with cell bodies positive for acetylated α-Tubulin; (ii) neurons containing LBs; (iii) neurons positive for acetylated α-Tubulin and containing LBs; (iv) neurons negative for both acetylated α-Tubulin and LBs. Despite the loss of neurons that characterizes the *substantia nigra*, the highest percentage of cells displaying acetylated α-Tubulin accumulation was in this region (Fig. 4a’). Furthermore, in all the areas analysed we found that acetylated α-Tubulin positive cells were ~40–70% (Fig. 4a’–c’), while neurons positive for acetylated α-Tubulin and containing LBs were uncommon (1–5 %; Fig. 4a’-c’), as confirmed by confocal microscopy (Fig. 4d–e”). Rarely we observed LBs positive for acetylated α-Tubulin (Fig. 4e-e”), whereas they were strongly positive for Ser129P α-Synuclein (Supplementary Fig. 5), a marker used for LBs and α-Synuclein pathology^65^. Taken together, these data suggest that neurons positive for acetylated α-Tubulin and those containing LB tend to be mutually exclusive.

**Fig. 5:**
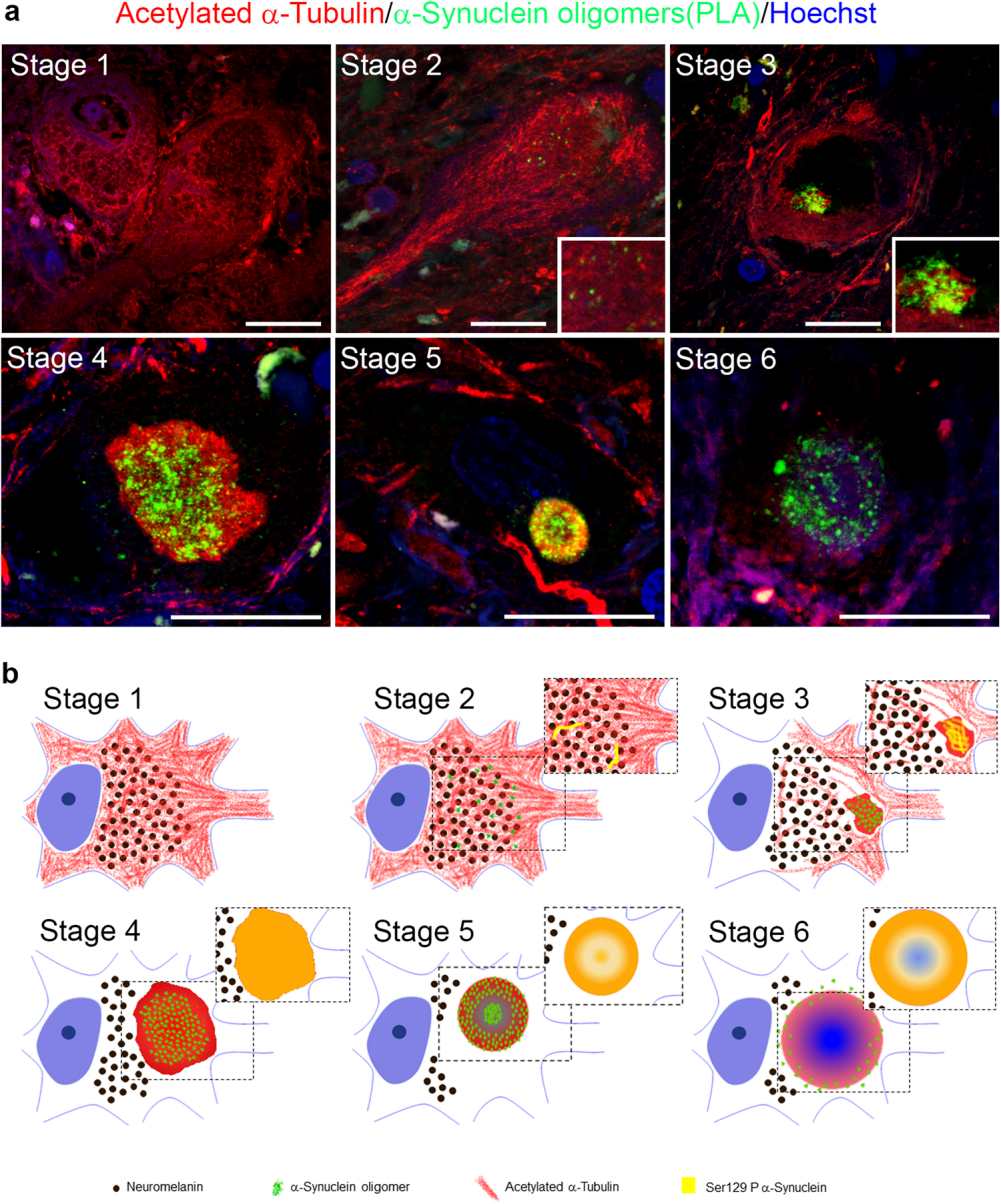
Linking acetylated α-Tubulin redistribution with α-Synuclein aggregation in Lewy body formation. **a** Six different stages are distinguishable in *post-mortem* human brain sections of PD patients following PLA assay for detecting α-Synuclein oligomers (green) and immunofluorescence for detecting acetylated α-Tubulin (red). **b** Schematic model showing the hypothetical sequences of events in LB formation starting from the redistribution of acetylated α-Tubulin (red) in the cell bodies of dopaminergic neurons to α-Synuclein oligomers (green) and Ser129 P α-Synuclein (yellow in the squared insets, see supplementary Fig. 6). Stage 1 shows the strong presence of acetylated α-Tubulin accumulated in the soma of neurons; in stage 2, acetylated α-Tubulin is still accumulated inside the cell body while some small and spared α-Synuclein oligomers and Ser129 P α-Synuclein appear; in stage 3, acetylated α-Tubulin starts to accumulate into a small aggregate with α-Synuclein oligomers and also Ser129 P α-Synuclein; stage 4 shows acetylated α-Tubulin in a bigger and compact aggregate with an undefined shape with α-Synuclein oligomers inside and strong Ser129 P α-Synuclein signal, whereas the cytoplasm is now negative for acetylated α-Tubulin staining; stage 5 shows a ring-shaped aggregate where acetylated α-Tubulin forms an external ring and α-Synuclein oligomers are distributed not only along the ring but also inside it. Ser129 P α-Synuclein has a pattern of distribution similar to α-Synuclein oligomers; in stage 6, the ring-shaped aggregate is weakly positive for acetylated α-Tubulin and has few α-Synuclein oligomers, while the Ser129 P α-Synuclein fibrils are still abundant in the external part. Hoechst staining (blue) is detectable in the core of aggregates at stage 6. Scale bar, 20 μm.

The presence of neurons displaying the enrichment of acetylated α-Tubulin in cell bodies and lacking mature LBs inside them, let us to hypothesize that the observed change in acetylated α-Tubulin could be involved in the initial step of α-Synuclein aggregation. We therefore focused on α-Synuclein oligomers^66^, the earliest α-Synuclein aggregated species^36,67,68^. Using super-resolution microscopy, we found a staging-like distribution between α-Synuclein and acetylated α-Tubulin. The combination between PLA to detect α-Synuclein oligomers and classical immunofluorescence for acetylated α-Tubulin allowed us to identify different stages that could describe LB formation (Fig. 5): stage 1, redistribution is already present as the cytoplasm of neurons is rich in acetylated α-Tubulin; stage 2, some α-Synuclein oligomers are spread in the cytoplasm in which acetylated α-Tubulin is widespread; stage 3, acetylated α-Tubulin starts to accumulate in small aggregates containing α-Synuclein oligomers; stage 4, acetylated α-Tubulin is entirely incorporated together with α-Synuclein oligomers inside the aggregate, whose shape is undefined; stage 5, inclusions are roundish with an external ring-shaped structure composed of acetylated α-Tubulin and α-Synuclein oligomers, which are also found in the centre of the structure; stage 6, α-Synuclein oligomers are localized mainly in the periphery of the aggregate, acetylated α-Tubulin staining is weak while diffuse Hoechst staining is detectable in the core region. To supply a more complete view on LB formation, we investigated also the relationship between acetylated α-Tubulin and Ser129 P α-Synuclein staining (Supplementary Fig. 6). Interestingly, Ser129 P α-Synuclein staining is detectable, although at low level, starting from the stage 2, it localizes inside small aggregates at stage 3, and strongly increases at stage 4. The staining for Ser129 P α-Synuclein becomes compact in the external part of the ring-shaped structure at stage 5, where also acetylated α-Tubulin is present, and in the inner part, where acetylated α-Tubulin is not detected. To note, α-Synuclein oligomers show a similar distribution of Ser129 P α-Synuclein at this stage (Fig. 5a). Finally, at stage 6, the staining of Ser129 P α-Synuclein remains intense in the external part of the aggregate, whereas acetylated α-Tubulin is almost absent and α-Synuclein oligomers are confined to the periphery (Fig. 5). Finally, we explored the relationship of acetylated α-Tubulin and α-Synuclein aggregation using a conformational antibody (clone 5G4,^69^), whose staining confirms our data of α-Synuclein oligomers and Ser129 P α-Synuclein in LB formation (Supplementary Fig. 7).

All these data highlight the involvement of acetylated α-Tubulin in the complex process of LB formation.

**Fig. 6:**
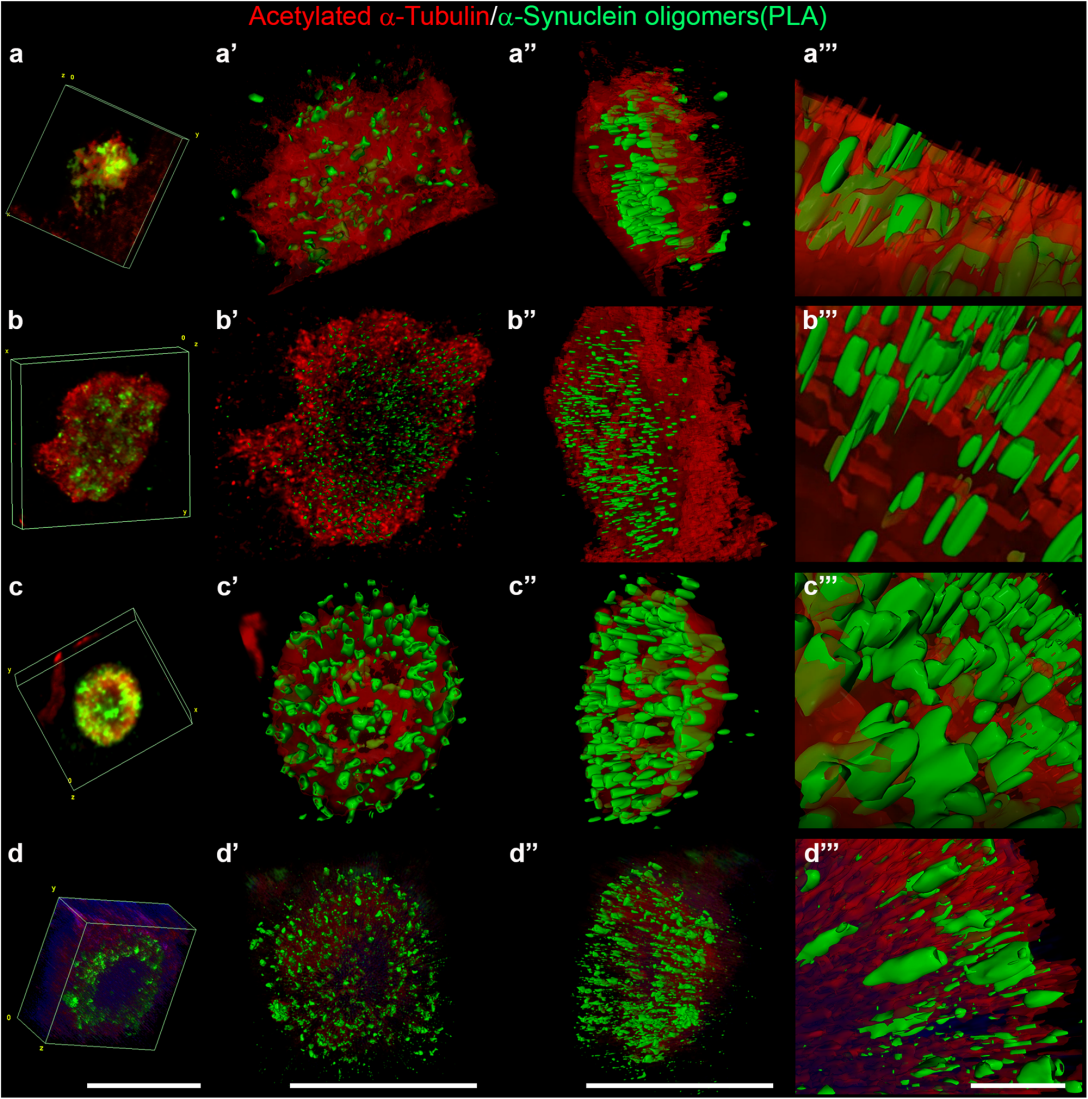
Representative 3D reconstruction of four stages of Lewy body formation (stage 3–6) obtained with confocal and arivis Vision4D® software. **a**–**a”’** stage 3; **b**–**b”’** stage 4; **c**–**c”’** stage 5; **d**–**d”’** stage 6. **a**, **b**, **c**, **d**: confocal 3D reconstruction obtained with Fiji software; **a’**, **b’**, **c’**, **d’**: frontal view of 3D reconstruction by arivis Vision4D® software; **a”**, **b”**, **c”**, **d”**: lateral view of 3D reconstruction by arivis Vision4D® software; **a”’**, **b”’**, **c”’**, **d”’**: magnifications by arivis Vision4D® software. **a**–**a”**, **b–b”**, **c**–**c”**, **d**–**d”**: scale bar, 20 μm; **a”’**, **b”’**, **c”’**, **d”’**: scale bar, 5 μm.

**Fig. 7:**
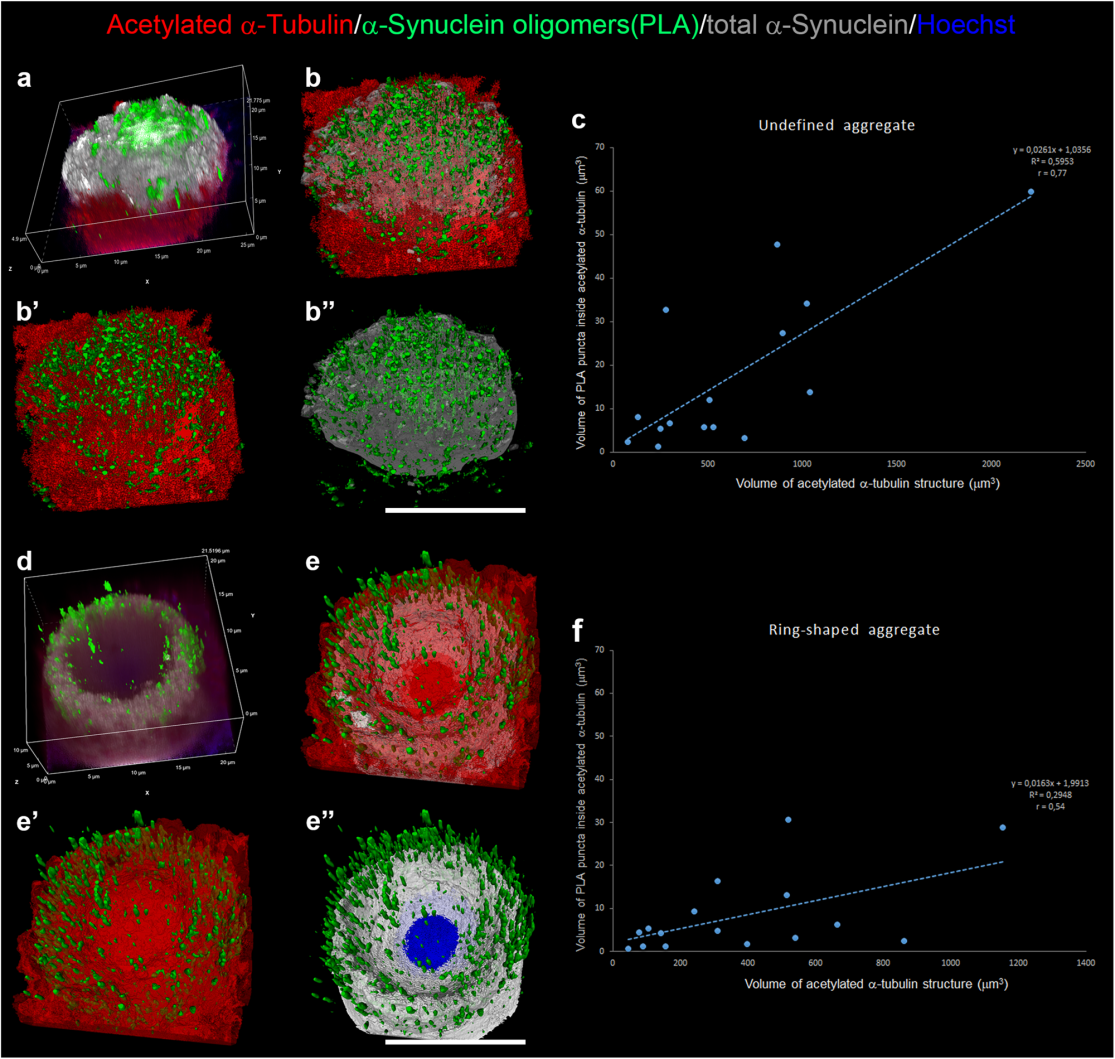
Correlation of α-Synuclein oligomers and acetylated α-Tubulin positive structures between undefined and ring-shaped aggregates. **a**, **b”** 3D reconstruction obtained with Nis Elements software (**a**) and arivis Vision4D® software (**b**–**b”**) of an undefined aggregate. Graph (**c**) shows the linear correlation between the volume of α-Synuclein oligomers and volume of acetylated α-Tubulin positive structures. **d**–**f** 3D reconstruction obtained with Nis Elements software (**d**) and arivis Vision4D® software (**e**–**e”**) of a ring-shaped aggregate. Graph (**f**) shows the linear correlation between the volume of α-Synuclein oligomers and the volume of acetylated α-Tubulin positive structures. Scale bar, 10 μm.

We further investigated the interplay between acetylated α-Tubulin and α-Synuclein oligomers by applying the imaging arivis Vision4D® software on high-resolution images (Figs. 6, 7; Supplementary Movie 1-4). This analytical programme guarantees precise and reproducible quantitative morphometric analyses^70–72^. This is particularly important for visualizing complex structures and their relationships, such as LB formation and the possible correlation between the acetylated α-Tubulin and α-Synuclein oligomers. Figure 6 reports representative images of aggregates from stage 3 to stage 6 that show 3D reconstructions obtained using a confocal microscope (Fig. 6a–d) and processed by arivis Vision4D® software (Fig. 6a’–a”’, b’–b”’, c’–c”’, d’–d”’). These analyses enabled us to explore in detail how α-Synuclein oligomers and acetylated α-Tubulin are distributed inside the different aggregates. Having confirmed the progressive changes in the localization of α-Synuclein oligomers inside the aggregates, we performed further in-depth measurements of PLA puncta in two main types of aggregates: (i) undefined aggregates, as observed in stages 3 and 4 (Fig. 7a–b”), and (ii) ring-shaped aggregates, as observed in stages 5 and 6 (Fig. 7d–e”). To better differentiate the two groups of aggregates, we also investigated total α-Synuclein staining (Fig. 7a, b”, d, e”). The average volume of PLA puncta was lower in undefined aggregates (0.029 ± 3.197 x 10^-6^ μm^3^, n. of PLA puncta = 26712 in 17 aggregates; Supplementary Fig. 8) compared to ring-shaped aggregates (0.045 ± 5.534 x 10^-6^ μm^3^, n. of PLA puncta = 17889 in 15 aggregates; Supplementary Fig. 8), which is in line with progressive α-Synuclein aggregation. Furthermore, we analysed the correlation between the volume of PLA puncta inside acetylated α-Tubulin positive structures and the acetylated α-Tubulin total volume. We found a correlation in both undefined (Fig. 7c; index correlation: 0.77) and ring-shaped (Fig. 7f; index correlation: 0.54) aggregates, supporting the hypothesis that the formation of acetylated α-Tubulin positive structures is strictly linked to α-Synuclein aggregation.

**Fig. 8:**
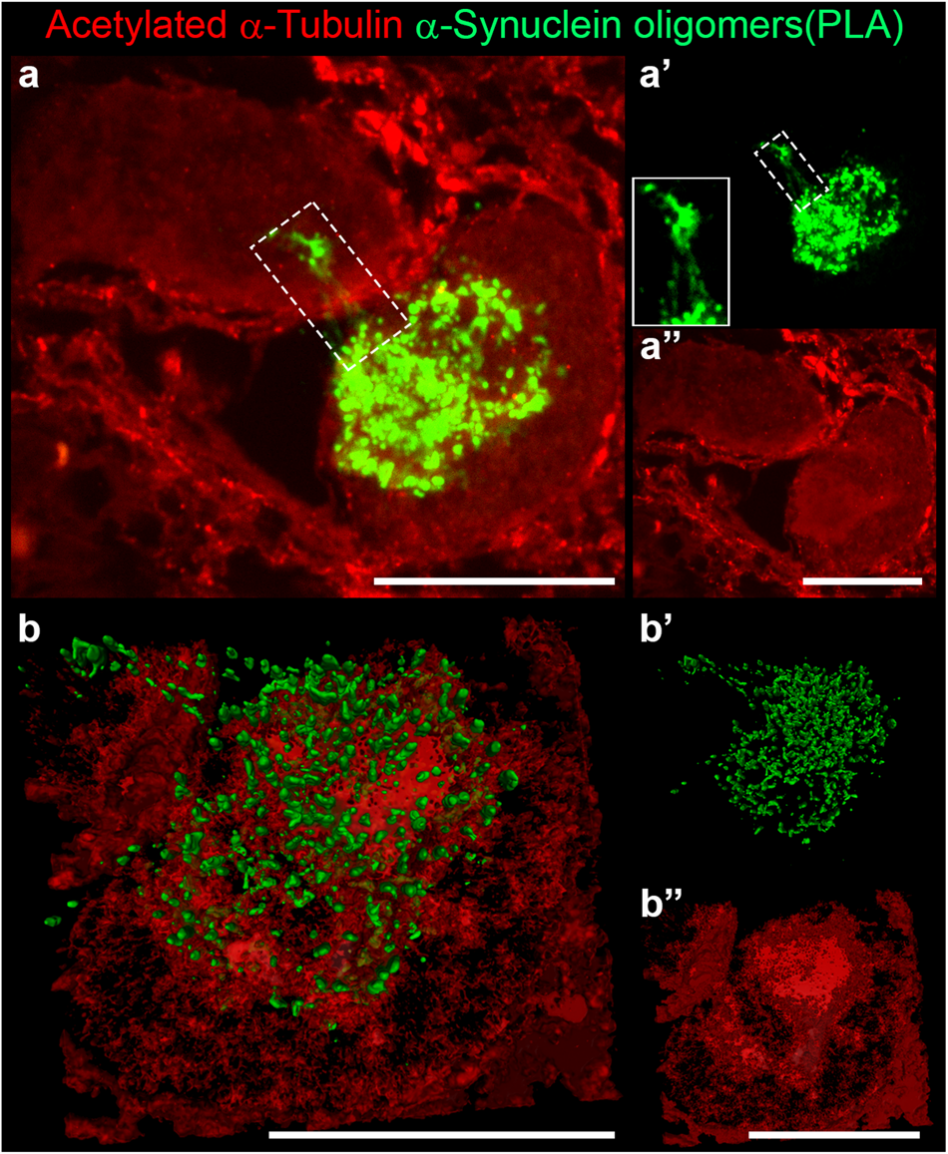
Tunnelling nanotube linking two neurons in *substantia nigra* of *post-mortem* human brain of PD patients. **a–a”** α-Synuclein oligomers (**a-a’**) and acetylated α-Tubulin (**a”**) mark the morphology of an undefined aggregate in neuronal cytoplasm. A tunnelling nanotube (white dashed rectangle, 2x magnified in **a’**) connects the two neurons and contains small oligomers. **b**–**b”** 3D reconstruction of the above confocal image with arivis Vision4D® software. Scale bar, 10 μm.

Finally, the extensive analysis of brain sections that we stained for α-Synuclein oligomers and acetylated α-Tubulin revealed a structure, that resembles the so-called tunnelling nanotube, previously described mainly in vitro in cellular models^37,39,40^, but never found in human brain. Indeed, we observed 6 threadlike structures in the *substantia nigra* detected by the presence of α-Synuclein oligomers in different patients. In detail, the tunnelling nanotubes are located between a neuron positive for acetylated α-Tubulin and another neuron (Fig. 8) or, alternatively, vessels and glial cells (Supplementary Fig. 9). 3D reconstruction enables us to better visualize the distribution of α-Synuclein oligomers in the sample (Fig. 8b–b”). The structures we identified could indicate the spreading of oligomers, confirming the diffusive properties of this species also in human brain.

## Discussion

This study explores the formation of α-Synuclein aggregates, referred to as the histopathological hallmark of PD, with the goal to understand the role of α-Tubulin in triggering neurodegenerative processes. Importantly, we performed these analyses on human tissues obtained from PD patients aware that this is the only way to understand the pathological processes that experimental models may not be able to fully recapitulate. We disclose the redistribution of acetylated α-Tubulin in *post-mortem* human brain of PD patients (as summarized in Fig. 3) and report its strong accumulation in neuronal cell bodies in the subcortical areas affected by LB pathology, from the dorsal motor nucleus of vagus to the *substantia nigra*^60^. Notably, acetylated α-Tubulin decreases in the fibres of both the putamen and skin sweat glands suggesting that its rearrangement could play a role both in the central and peripheral nervous system. Hence, we linked the redistribution of acetylated α-Tubulin with α-Synuclein pathology and found that the accumulation of acetylated α-Tubulin in the cell soma precedes the formation of mature LBs, as revealed by the detailed analysis of the earliest forms of α-Synuclein aggregation i.e. oligomers. Starting from the staging of LB maturation previously proposed^42,46,73^, we unravelled a correlation between the redistribution of acetylated α-Tubulin and the formation of α-Synuclein oligomers as well as Ser129 P α-Synuclein. This supplies further evidence to the emerging picture of LB formation, in which acetylated α-Tubulin may have an active part and play a driving-role.

To date, the evidence for a functional role of α-Tubulin acetylation is emerging in acute and toxic or gene-based models of parkinsonism^74^. An overall increase in α-Tubulin acetylation marks neuronal-like cells exposed to the neurotoxin MPP^+^^27^ and is detectable in dopaminergic fibres of the corpus striatum and in cell bodies of the *substantia nigra* dopaminergic neurons in mice injected with the neurotoxin MPTP^29^. Similarly, neuroblastoma cells treated with 6-hydroxydopamine (6-OHDA) display an increase in acetylated α-Tubulin due to a reduction in the tubulin deacetylase enzyme SIRT2^75^. Interestingly, such an imbalance in the level of modified tubulin seems to precede the impairment of mitochondria transport in MPP^+^-treated cells^27^. Thus, these studies converge on the hypothesis that increased acetylation of α-tubulin is implicated in the mechanisms of neurodegeneration. As regards genes linked to familial and non-familial forms of PD, including *PARK2* (encoding for Parkin) and *LRRK2* (encoding for Leucine-rich repeats kinase 2, LRRK2), the interplay between changes in acetylation of α-tubulin and functional defects in axonal transport is evident^48,76^. Beyond the known role of Parkin in the stabilization of microtubules^77,78^, it has been reported that in the *PARK2* KO mouse model, acetylated α-tubulin has enhanced in dopaminergic neurons and fibres, and precedes the impairment of axonal mitochondria transport, as revealed by the clustering of mitochondria along the axons^79^. To note, in dopaminergic neurons obtained from iPSCs, derived from patients carrying *PARK2* mutations, acetylated α-Tubulin is altered and displays a discontinuous and gapped staining on microtubules suggesting their overall weakness and dysfunction^79^. Furthermore, primary fibroblasts obtained from skin biopsies carrying the *LRRK2* mutation (p.G2019S) display an increase in acetylated α-Tubulin in their cellular bodies^80^. In contrast, Godena and colleagues demonstrated that LRRK2 pathogenic forms induce a decrease in acetylated α-tubulin, impairing and disrupting mitochondrial transport in cortical neurons and motor deficits in *D. melanogaster*, which are rescued increasing the acetylation on microtubules^81^. Further to these debated results obtained in vitro and in animal models, to date, the only evidence that the human brain experiences an imbalance of acetylated α-Tubulin in PD lies on biochemical analyses performed on the *substantia nigra pars compacta*^30^. In this study, the level of α-Tubulin acetylation is significantly decreased in the *substantia nigra* of PD patients whereas it remains unchanged in the hippocampus and in the cortex. Using in-situ analyses, we demonstrate, that a redistribution of acetylated α-Tubulin occurs in patients affected with idiopathic PD. Indeed, we unravelled the significant accumulation of acetylated α-Tubulin in neuronal cell bodies of subcortical regions whereas no significant change was observed in cortical areas. To note, the area-specific alterations that we unravelled are concordant with data of Esteves et al.^30^. In addition, the apparent discrepancy observed in Esteves’ and our work concerning the decrease of acetylated α-Tubulin in PD, is due to the different techniques applied. Using immunohistochemistry, we focused only on neurons, and, in detail, on neuronal cell body. Instead, using Western blotting, a cell type-related analysis cannot be performed. For this reason, the two studies are not in contrast. Surely, we cannot exclude that the decrease of acetylated α-Tubulin revealed by biochemical data could be due to an impairment in the tangle neuropil of *substantia nigra pars compacta*. Finally, our data shed light on the emerging role of acetylated α-Tubulin as a reliable player in neurodegeneration processes underlying PD.

Our study first demonstrates that although the increase in the number of neuronal cell bodies positive for acetylated α-Tubulin, neurons containing mature LBs are rarely positive for acetylated α-Tubulin. This could be due to the time-course of the events, in which the enrichment of acetylated α-Tubulin occurs in an early temporal window compared to the appearance of the final aggregated form of α-Synuclein. Our analysis of α-Synuclein oligomers, which are suggested to be the earliest and most toxic species, at least in the context of the membrane damage, of α-Synuclein aggregates^66,67,82^ allowed us to explore in detail the formation of LBs and to propose a model of staging in which acetylated α-Tubulin and α-Synuclein oligomers or small aggregates are involved (Figs. 5b and 7b). To date, in the sequence of events that leads to LB formation, α-Synuclein is first diffuse in the cytoplasm, gradually accumulates into irregularly-shaped aggregates, and, finally, into well-defined round-shaped inclusions such as pale bodies and LBs^43,44^. The complexity of LB formation is very difficult to be recapitulated in cell or animal models and the histological analysis of brain section from *post-mortem* PD patients was proved to be crucial for the investigation of both the composition (more than 100 proteins have been localized inside it) and the morphological aspects of LB^42^. The proteins detected has been described to be involved in different cellular processes, such as protein degradation, vesicular transport, oxidative stress, signal transduction, synaptic transmission and apoptosis^45,83–87^. Besides protein also lipids, as membranous fragments, organelles and vesicles characterize LB biogenesis^45,87,88^. These data lead to a staging of LB formation^42^. Very recently, Moors and colleagues confirmed a staging and revealed details as to the framework of neurofilaments, α-Tubulin, and phosphorylated α-Synuclein inside the aggregates using STED microscopy^46^. Our study combined the PLA approach to detect α-Synuclein oligomers and immunofluorescence for acetylated α-Tubulin with spinning disk confocal microscopy, SoRa super-resolution, and 3D-reconstruction, to propose a multistage model for LB formation. This model involves acetylated α-Tubulin starting from stage 1, where it accumulates in the neuronal cell bodies prior to the appearance of α-Synuclein oligomers and Ser129 P α-Synuclein, to stage 6, where oligomers are localised mainly at the periphery of the inclusion, Ser129 P α-Synuclein is strongly present in the external ring, as expected, and staining for acetylated α-Tubulin is weak (Fig. 7b). The initial increase in α-Tubulin acetylation could be explained by defects in the activity of the enzymes that regulate acetylation on Lys40 of α-Tubulin, namely α-TAT1^89,90^ or its deacetylation, namely HDAC6^91,92^. However, we observed the decrease of acetylated α-Tubulin in the cytoplasm during LB formation. We hypothesise that α-Synuclein might bind and sequester acetylated α-Tubulin thus reducing its level in the cytoplasm or, on the contrary, that accumulating acetylated α-Tubulin drives α-Synuclein oligomerization and aggregation into LBs. Our data showing the correlation between the volume of PLA puncta (i.e. α-Synuclein oligomers), and acetylated α-Tubulin (Fig. 7c, f) point to the driving role of acetylated α-Tubulin. In stage 6, acetylated α-Tubulin is reduced inside ring-shaped aggregates (Fig. 7a). This decrease in acetylated α-Tubulin could be due by an increase of α-Tubulin deacetylation, by its degradation or rather by the difficulties of the antibody to reach its epitope, if buried inside the dense inclusion. Based on previous work indicating that the main tubulin deacetylated enzyme, HDAC6, is present with its active form (i.e. P-HDAC6) in the LBs of different parkinsonism, including PD^57^, our preferred hypothesis is the increase of Tubulin deacetylation. In support of this, we found that acetylated α-Tubulin is more evident in the outer part of the aggregate (Supplementary Fig. 10), while P-HDAC6 is mainly concentrated in the inner part, where acetylated α-Tubulin is less visible. Based on this, we can speculate that HDAC6 present inside the LB de-acetylates α-Tubulin causing the lack of acetylated α-Tubulin staining. Our research on LBs formation adds a piece to a puzzle whose building started since their discovery. In particular, biogenesis of LBs has been also related to aggresomes, which are cytoprotective inclusions involved in the degradation of accumulated proteins^93^. HDAC6 is implicated in the aggresome formation for the transport of ubiquitinated misfolded proteins through the microtubule network^94^. This link with HDAC6 could indicate a protective role for acetylated α-Tubulin. However, the increase of acetylated α-Tubulin in the neuronal cell body begins before LB formation. For this reason, the question remains whether the increase of acetylated α-Tubulin is protective or detrimental for neurons. The inhibition of HDAC6 with Tubastatin A in a rat model of PD-like neurodegeneration increases acetylated α-Tubulin and protects dopaminergic neurons reducing not only α-Synuclein toxicity and its phosphorylation at Ser129 but also neuroinflammation^95^. In addition, Outeiro and colleagues revealed that the genetic and pharmacological inhibition of the other deacetylase of acetylated α-tubulin, SIRT2^92^, increases acetylated α-tubulin in cell models and reduces α-Synuclein aggregation and toxicity^96^. Finally, studies on PD cybrid cells revealed that davunetide (NAP), a peptide promoting microtubule assembly, is able to reduce α-Synuclein oligomerization and restore microtubule-dependent trafficking by increasing the levels of acetylated α-Tubulin^97^. Very recently, we showed that treatment of HDAC6 with a specific inhibitor, Tubacin, leads to acetylated α-Tubulin accumulation and, in turn, to an increase of oligomeric α-Synuclein in cell model overexpressing α-Synuclein^98^. Hence, although data from experimental models suggest that acetylated α-tubulin control could be a promising strategy to reduce α-Synuclein aggregation and ameliorate PD conditions, its mechanism, namely reduction or stimulation, remains a matter of debate.

Interestingly, we report in *post-mortem* human brains the presence of threadlike structures that contain α-Synuclein oligomers and resemble tunnelling nanotubes. Until now, these structures have been seen exclusively in in vitro models. Tunnelling nanotubes were visualised between a donor and an acceptor neuron in co-cultures of mouse neuronal-like cells and were composed of α-Synuclein fibrils in lysosomal vesicles that can seed α-Synuclein aggregation in the acceptor cell^39^. More recently, another study elucidated the mechanism of α-Synuclein spreading with tunnelling nanotubes showing that α-Synuclein is able to modify the function of lysosomes and use these organelles to propagate and spread between cells^99^. Moreover, Valdinocci and colleagues pointed out that α-Synuclein can bind migrating mitochondria within tunnelling nanotubes to increase its aggregation in mono and co-cultures of macrophages and neuroblastoma cells^100^. Our study provides evidence that tunnelling nanotubes could be a strategy used by neurons in the central nervous system for the spreading of α-Synuclein pathology and, also interestingly, that acetylated α-Tubulin could be involved in the tunnelling nanotube formation. This hypothesis is based on the observation that the neurons associated with the threadlike structure containing α-Synuclein oligomers have increased acetylated α-Tubulin.

The neuronal imbalance of acetylated α-Tubulin is not exclusive to PD, but has been demonstrated in many neurodegenerative diseases^74^. In detail, changes in acetylated α-Tubulin have been reported in experimental models of Charcot-Marie-Tooth disease^101,102^, Amyotrophic Lateral Sclerosis^103,104^, Huntington’s^105^ and Alzheimer’s disease^106^. As regards *post-mortem* human brain, an imbalance of acetylated α-Tubulin is recorded in patients affected by neurodegenerative disease. In *post-mortem* striatal samples of Huntington’s disease (grade 3/4), Western blotting analysis revealed a dramatic reduction in acetylated α-Tubulin compared to controls, while total α-Tubulin levels were unaffected^105^. A recent study on *post-mortem* human brain of Alzheimer’s disease patients at Braak stage III-IV reports a decrease in the level of acetylated α-Tubulin in the cortex and hippocampus^30^. A previous work reported both a decrease in acetylated α-Tubulin and a reduction of total α-Tubulin in the hippocampus of Alzheimer’s disease patients compared to controls, with a net increase in the acetylation status of α-Tubulin^107^. However, the implication of acetylated α-Tubulin in Alzheimer’s disease remains controversial as recent evidence shows that it accumulates in *post-mortem* brain tissues from patients^108^. Interestingly, Zhang and colleagues reported the accumulation of acetylated α-Tubulin in cell bodies of hippocampal neurons and this is similar to what we observed in PD brain and have described in our study^107^. Thus, our data open the way to investigate how the redistribution of this modified form of Tubulin, which is crucial for the maintenance of the proper mechanical properties of microtubules, could impact the susceptibility of specific anatomical areas to neurodegeneration in PD and beyond.

In conclusion, this study reveals the contribution of acetylated α-Tubulin to LB formation in *post-mortem* human brains affected by PD. The completely unexplored link between the redistribution of acetylated α-Tubulin and α-Synuclein oligomerization in the human brain definitively supplies a perspective for investigating the drivers of pathological aggregation in PD.

## Methods

### Patients, clinical and neuropathological assessment

All the patients were enroled in the study and followed during the course of their disease by neurologists experienced in movement disorders and dementia at the ASST G. Pini-CTO Parkinson’s Centre in Milan. Written informed consent was obtained from all subjects prior to enrolment. Clinical diagnosis of PD was established according to the UK Brain Bank criteria^109,110^. *Post-mortem* human brains were collected by the Nervous Tissues Bank (Milan, Italy) and clinical diagnosis was confirmed by neuropathological analysis, according to the current BrainNet Europe Consortium guidelines^111^ by two experts, GG and MB. *Post-mortem* human brains obtained from patients fulfilling clinical and neuropathological diagnostic criteria for PD (*N* = 12) and from age- and sex-matched healthy subjects (*N* = 6) were used. Demographic data, disease duration and disease severity (according to the Hoehn and Yahr staging system^112^) are shown in Supplementary Table 1. The study procedures were in accordance with the principles outlined in the Declaration of Helsinki and approved by the Ethics Committee of the University of Milan (protocol code 66/21). Skin biopsies were collected from healthy subjects (*N* = 9) and PD patients (*N* = 12) by the Parkinson Institute Biobank^113^ (Supplementary Table 2).

### Human brain samples

Human brains were obtained at autopsy and fixed in 10% buffered formalin for at least 21 days. Specimens from bulb, pons, mesencephalon, basal ganglia, entorhinal, cingulate and the frontal cortex of both controls and PD subjects were embedded in paraffin and then cut into 5-μm thick sections using a microtome (MR2258, Histoline) and processed as follows: (i) one slice was stained with haematoxylin and eosin to verify the tissue morphology; (ii) one slice was stained for α-Synuclein to verify or exclude its accumulation in LBs and to assess its relative staging, and (iii) the sections underwent further immunohistochemistry and immunofluorescence assay for comparative localization of the different antigens.

### Skin biopsies

Volar forearm skin biopsies were fixed in Zamboni solution for 24 h at 4 °C and processed as previously described^64^. Briefly, the paraffin embedded samples were sliced into 3-μm thick serial sections and processed: (i) for histology (haematoxylin and eosin to verify the presence of sweat glands); (ii) immunohistochemistry to evaluate the presence of α-Synuclein in the synaptic terminals; and (iii) immunofluorescence assay for comparative localization of the different antigens. In all these samples, we previously checked the presence of α-Synuclein oligomers^64^.

### Immunohistochemistry

After deparaffination and rehydration, brain sections were sequentially incubated with (i) 3% H_2_O_2_ for 20 min; (ii) 1% BSA diluted in 0.01M phosphate saline buffer (PBS) containing 0.1% Triton™ X-100 (BSAT) for 20 min; (iii) the primary mouse anti-acetylated α-Tubulin antibody (clone 6-11B-1), rabbit anti-acetylated α-Tubulin antibody (clone D20G3) or mouse anti-α-Tubulin antibody, overnight at room temperature (RT). Antigen-antibody interaction was visualized using EnVision™ anti-mouse or anti-rabbit secondary antibody conjugated with HRP (1 h at RT), and 3,3′ diaminobenzidine as a chromogen (DAB; Dako kit).

In order to assess the comparative localization of acetylated α-Tubulin with α-Synuclein, we performed double immunoenzymatic staining using adjacent sections containing *substantia nigra*, nucleus basalis of Meynert and entorhinal cortex. After incubation with 20% acetic acid for 20 min to inactivate endogenous alkaline phosphatase (AP), the samples were incubated with a mix of mouse anti-acetylated α-Tubulin and rabbit anti α-Synuclein antibody (S3062, aa 111-132). To visualize double immunostaining, we used: (i) anti rabbit ImmPRESS™-AP secondary antibody conjugated with AP and a substrate solution of 1-Naphthyl phosphate disodium salt (1 mg/ml) and Fast Blue B salt (1 mg/ml) dissolved in 0.1 M Tris-HCl (pH 9.2–9.4) for α-Synuclein; and (ii) EnVision™ anti-mouse and DAB for acetylated α-Tubulin.

Although we used the well-characterized mouse monoclonal 6-11B-1 for the staining of acetylated α-tubulin^114^, we checked for its specificity on brain samples. First, primary antibody omission was performed. Next, anti-acetylated α-Tubulin antibody pre-adsorbed with an excess of tubulin (antibody: tubulin 1:5) and an immunoenzymatic assay on *post-mortem* human brain samples (Supplementary Fig. 11). The tubulin necessary for these experiments was obtained in our laboratory from a healthy bovine brain following a well-established protocol^115^, and an additional chromatographic run using a MonoQ GL column (GE Healthcare Life Sciences). Finally, tubulin was eluted in BRB80 buffer with 1 M KCl, 0.1 mM GTP, 0.1 mM PMSF, 0.1 mM DTT and analysed by SDS-PAGE and western blotting to verify the presence of acetylated α-Tubulin.

### Immunofluorescence and Proximity Ligation Assay

Tissue sections containing *substantia nigra*, basal ganglia, frontal cortex and skin biopsies were used for classical immunofluorescence. The tissues were incubated with BSAT for 20 min at RT followed by a mixture of anti-acetylated α-Tubulin (6-11B-1 or D20G3) with different primary antibodies (TH, S100β, MBP, IBA1, MAP2, Tau, α-Synuclein S3062, Ser129 P α-Synuclein, aggregated α-Synuclein 5G4, α-Tubulin, PGP 9.5, synaptophysin; see Supplementary Table 3 for details on antibodies) overnight at RT, followed by incubation with highly pre-adsorbed secondary antibodies (see Supplementary Table 3 for details on antibodies) for 2 h at RT in the dark.

We used PLA to detect α-Synuclein oligomers, as previously described^64^. Briefly, mesencephalic sections were incubated with a mixture containing α-Synuclein S3062-MINUS, α-Synuclein S3062-PLUS probes, and anti-acetylated α-Tubulin antibody in PLA diluent for 2 h at 37 °C and overnight at RT. The amplification reaction was performed in serial incubation steps: (i) ligase in Duolink® ligation solution for 1 h at 37 °C, and (ii) polymerase in Duolink® amplification reagent for 2 h at 37 °C, to which donkey anti-mouse secondary antibody was added. Nuclei were stained using Hoechst 33342 (10 min RT) and the samples were mounted using Mowiol-DABCO®.

### Microscopy and 3D reconstruction

The sections were analysed with a Nikon spinning disk confocal microscope using a water-immersion 40x objective. In addition, we used a spinning disk super-resolution by optical pixel reassignment (SoRa) technique using the silicon-immersion 100x objective plus a resolution improvement of 2.8x. Images were analysed with Fiji software (NIH). For 3D visualisation, images were imported into arivis Vision4D® software (Zeiss Company) containing all the z-stacks in their native format. The images were transformed into the 12-pixel format and region of interest (ROI) were selected using the “Transformation gallery > Crop” tool. Images contain 4 colour channels: Hoechst in blue, acetylated α-Tubulin in red, α-Synuclein oligomers in green and total synuclein in white. The “Intensity threshold segmentation” pipeline was used for the blue and the red signals. The PLA signal showed a scattered and pointy distribution, so the “Blob Finder” pipeline was chosen and using “preview”, a suitable range of exposure was selected. As staining was strong and continuous in a compact area, “Machine Learning Segmentation” was chosen as a suitable pipeline for total-α-Synuclein. Finally, the analysis was run from “Run analysis pipeline” and the reconstruction was visualized via 4D view. The analysis was then carried out using a “cell or particle compartmentalization pipeline”, which can find partial or total overlapping conditions between two reconstructed objects and can be applied to any kind of small particles.

### Data and statistical analysis

The data were gathered using Fiji software (NIH). In detail, Fiji tools were used to measure areas, cell counter for counting cells in each anatomic region, and JACOP plug-in for co-localization analysis calculating Mander’s coefficient^116^. For skin, sweat glands confocal images (40x magnification) were reconstructed in a z-stack and analysed with Fiji software (NIH). We conducted the quantitative analysis in the specimens in all the sweat glands (*n* = 11 for controls; *n* = 18 for patients). The acetylated α-Tubulin staining was rated as the area of acetylated α-Tubulin signal within PGP 9.5 positive fibres, normalized for the area of PGP 9.5 positive fibres. In particular, the localization of acetylated α-Tubulin signal inside fibres was assessed by the superimposition of the mask of the PGP 9.5-positive green channel and the acetylated α-Tubulin-positive red channel.

Statistical analysis was carried out using GraphPad Prism 8. It was performed the Mann-Whitney test, two-tailed, being the distribution of the experimental data not normal, accordingly to the normality tests (D’Agostino and Pearson test and Shapiro-Wilk test). A *p*-value < 0.05 was considered statistically significant.

In the images obtained using arivis Vision4D® software, the signal was transformed into an object. Data collected from the “object table” by exporting “analysis objects” were the volume of all PLA puncta, the volume of all acetylated α-Tubulin positive objects, the volume of the PLA puncta inside acetylated α-Tubulin positive objects, the volume of the PLA puncta intersecting at least 1% acetylated α-Tubulin positive objects, the mean volume, and the total number of each of the previously listed objects. These data were analysed using frequency distribution and linear correlation, obtaining Spearman’s rank correlation coefficients (ρ).

### Reporting summary

Further information on research design is available in the Nature Research Reporting Summary linked to this article.

### Supplementary information

Supplementary information

Reporting Summary

Supplementary Movie 2

Supplementary Movie 3

Supplementary Movie 4

Supplementary Movie 1

## Data Availability

The datasets generated and/or analyzed during the current study, and the arivis Vision 4D software pipelines generated by for image analyses, are available from the corresponding authors.

## References

[1] Muñoz-Lasso DC, Romá-Mateo C, Pallardó FV, Gonzalez-Cabo P (2020). Much more than a scaffold: cytoskeletal proteins in neurological disorders. Cells.

[2] Rolls MM, Thyagarajan P, Feng C (2021). Microtubule dynamics in healthy and injured neurons. Dev. Neurobiol..

[3] Waites C, Qu X, Bartolini F (2021). The synaptic life of microtubules. Curr. Opin. Neurobiol..

[4] Kapitein LC, Hoogenraad CC (2015). Building the neuronal microtubule cytoskeleton. Neuron.

[5] Sferra A, Nicita F, Bertini E (2020). Microtubule dysfunction: a common feature of neurodegenerative diseases. Int J. Mol. Sci..

[6] Cappelletti, G. & Cartelli, D. Acetylation of tubulin: A feasible protective target from neurodevelopment to neurodegeneration. in *Neuroprotection in Autism, Schizophrenia and Alzheimer’s Disease* 273–294 (Elsevier, 2020).

[7] Cappelletti G (2015). Linking microtubules to Parkinson’s disease: the case of parkin. Biochem Soc. Trans..

[8] Matamoros AJ, Baas PW (2016). Microtubules in health and degenerative disease of the nervous system. Brain Res. Bull..

[9] Brandt R, Bakota L (2017). Microtubule dynamics and the neurodegenerative triad of Alzheimer’s disease: the hidden connection. J. Neurochem..

[10] Fanara P (2007). Stabilization of hyperdynamic microtubules is neuroprotective in amyotrophic lateral sclerosis. J. Biol. Chem..

[11] Janke C, Magiera MM (2020). The tubulin code and its role in controlling microtubule properties and functions. Nat. Rev. Mol. Cell Biol..

[12] Gadadhar S, Bodakuntla S, Natarajan K, Janke C (2017). The tubulin code at a glance. J. Cell Sci..

[13] Song Y, Brady ST (2015). Post-translational modifications of tubulin: pathways to functional diversity of microtubules. Trends Cell Biol..

[14] L’Hernault SW, Rosenbaum JL (1983). Chlamydomonas alpha-tubulin is posttranslationally modified in the flagella during flagellar assembly. J. Cell Biol..

[15] Creppe C (2009). Elongator controls the migration and differentiation of cortical neurons through acetylation of α-Tubulin. Cell.

[16] Nekooki-Machida Y, Hagiwara H (2020). Role of tubulin acetylation in cellular functions and diseases. Med. Mol. Morphol..

[17] Janke C, Kneussel M (2010). Tubulin post-translational modifications: encoding functions on the neuronal microtubule cytoskeleton. Trends Neurosci..

[18] Portran D, Schaedel L, Xu Z, Théry M, Nachury MV (2017). Tubulin acetylation protects long-lived microtubules against mechanical ageing. Nat. Cell Biol..

[19] Xu Z (2017). Microtubules acquire resistance from mechanical breakage through intralumenal acetylation. Science.

[20] Montagnac G (2013). TAT1 catalyses microtubule acetylation at clathrin-coated pits. Nature.

[21] Geeraert C (2010). Starvation-induced hyperacetylation of tubulin is required for the stimulation of autophagy by nutrient deprivation. J. Biol. Chem..

[22] Reed NA (2006). Microtubule acetylation promotes kinesin-1 binding and transport. Curr. Biol..

[23] Aguilar A (2014). Tubulin K40 acetylation is required for contact inhibition of proliferation and cell–substrate adhesion. Mol. Biol. Cell.

[24] Hunn BHM, Cragg SJ, Bolam JP, Spillantini M-G, Wade-Martins R (2015). Impaired intracellular trafficking defines early Parkinson’s disease. Trends Neurosci..

[25] Pellegrini L, Wetzel A, Grannó S, Heaton G, Harvey K (2017). Back to the tubule: microtubule dynamics in Parkinson’s disease. Cell. Mol. Life Sci..

[26] Cartelli D, Cappelletti G (2017). Microtubule destabilization paves the way to Parkinson’s disease. Mol. Neurobiol..

[27] Cartelli D (2010). Microtubule dysfunction precedes transport impairment and mitochondria damage in MPP+-induced neurodegeneration. J. Neurochem..

[28] Kim-Han JS, Antenor-Dorsey JA, O’Malley KL (2011). The Parkinsonian mimetic, MPP+, specifically impairs mitochondrial transport in dopamine axons. J. Neurosci..

[29] Cartelli D (2013). Microtubule alterations occur early in experimental parkinsonism and the microtubule stabilizer epothilone D is neuroprotective. Sci. Rep..

[30] Esteves AR, Cardoso SM (2020). Differential protein expression in diverse brain areas of Parkinson’s and Alzheimer’s disease patients. Sci. Rep..

[31] Panicker, N., Ge, P., Dawson, V. L. & Dawson, T. M. The cell biology of Parkinson’s disease. *J. Cell Biol.* **220**, e00241 (2021). 10.1083/jcb.202012095 PMC8103423 33749710

[32] Balestrino R, Schapira AHV (2020). Parkinson disease. Eur. J. Neurol..

[33] Poewe W (2017). Parkinson disease. Nat. Rev. Dis. Prim..

[34] Dickson DWParkinson’s (2012). Disease and Parkinsonism: neuropathology. Cold Spring Harb. Perspect. Med..

[35] Spillantini MG (1997). Synuclein in Lewy bodies. Nature.

[36] Bengoa-Vergniory N, Roberts RF, Wade-Martins R, Alegre-Abarrategui J (2017). Alpha-synuclein oligomers: a new hope. Acta. Neuropathol..

[37] Valdinocci D, Radford R, Siow S, Chung R, Pountney D (2017). Potential modes of intercellular α-Synuclein transmission. Int. J. Mol. Sci..

[38] Uemura N, Uemura MT, Luk KC, Lee VM-Y, Trojanowski JQ (2020). Cell-to-cell transmission of Tau and α-Synuclein. Trends Mol. Med.

[39] Abounit S (2016). Tunneling nanotubes spread fibrillar α‐synuclein by intercellular trafficking of lysosomes. EMBO J..

[40] Mao X (2016). Pathological α-synuclein transmission initiated by binding lymphocyte-activation gene 3. Science.

[41] Danzer KM (2012). Exosomal cell-to-cell transmission of alpha synuclein oligomers. Mol. Neurodegener..

[42] Fares MB, Jagannath S, Lashuel HA (2021). Reverse engineering Lewy bodies: how far have we come and how far can we go?. Nat. Rev. Neurosci..

[43] Takahashi H, Wakabayashi K (2001). The cellular pathology of Parkinson’s disease. Neuropathology.

[44] Wakabayashi K (2013). The lewy body in Parkinson’s disease and related neurodegenerative disorders. Mol. Neurobiol..

[45] Shahmoradian SH (2019). Lewy pathology in Parkinson’s disease consists of crowded organelles and lipid membranes. Nat. Neurosci..

[46] Moors TE (2021). The subcellular arrangement of alpha-synuclein proteoforms in the Parkinson’s disease brain as revealed by multicolor STED microscopy. Acta. Neuropathol..

[47] Seebauer L (2022). Interaction of alpha synuclein and microtubule organization is linked to impaired neuritic integrity in Parkinson’s patient-derived neuronal cells. Int. J. Mol. Sci..

[48] Calogero AM, Mazzetti S, Pezzoli G, Cappelletti G (2019). Neuronal microtubules and proteins linked to Parkinson’s disease: a relevant interaction?. Biol. Chem..

[49] Cartelli D (2016). Synuclein is a novel microtubule dynamase. Sci. Rep..

[50] Payton JE, Perrin RJ, Clayton DF, George JM (2001). Protein–protein interactions of alpha-synuclein in brain homogenates and transfected cells. Mol. Brain Res..

[51] Alim MA (2002). Tubulin seeds α-Synuclein fibril formation. J. Biol. Chem..

[52] Alim MA (2004). Demonstration of a role for α-synuclein as a functional microtubule-associated protein. J. Alzheimer’s Dis..

[53] Zhou RM (2010). Molecular interaction of α-synuclein with tubulin influences on the polymerization of microtubule in vitro and structure of microtubule in cells. Mol. Biol. Rep..

[54] Amadeo A (2021). The association between α-synuclein and α-tubulin in brain synapses. Int. J. Mol. Sci..

[55] Cartelli D, Cappelletti G (2017). α-Synuclein regulates the partitioning between tubulin dimers and microtubules at neuronal growth cone. Commun. Integr. Biol..

[56] Lee H, Zhu X, Takeda A, Perry G, Smith MA (2006). Emerging evidence for the neuroprotective role of α-synuclein. Exp. Neurol..

[57] Mazzetti, S. et al. Phospho-HDAC6 gathers into protein aggregates in Parkinson’s dsease and a typical Parkinsonisms. *Front. Neurosci.* **14**, 784054 (2020). 10.3389/fnins.2020.00624 PMC7324673 32655357

[58] Liddelow SA (2017). Neurotoxic reactive astrocytes are induced by activated microglia. Nature.

[59] Dheen TS, Kaur C, Ling E-A (2007). Microglial activation and its implications in the brain diseases. Curr. Med. Chem..

[60] Braak H, Ghebremedhin E, Rüb U, Bratzke H, Del Tredici K (2004). Stages in the development of Parkinson’s disease-related pathology. Cell Tissue Res..

[61] Klingelhoefer L, Reichmann H (2017). Parkinson’s disease as a multisystem disorder. J. Neural Transm..

[62] Zange L, Noack C, Hahn K, Stenzel W, Lipp A (2015). Phosphorylated α-synuclein in skin nerve fibres differentiates Parkinson’s disease from multiple system atrophy. Brain.

[63] Donadio V (2016). Skin nerve misfolded α-synuclein in pure autonomic failure and Parkinson disease. Ann. Neurol..

[64] Mazzetti S (2020). α-Synuclein oligomers in skin biopsy of idiopathic and monozygotic twin patients with Parkinson’s disease. Brain.

[65] Fujiwara H (2002). Synuclein is phosphorylated in synucleinopathy lesions. Nat. Cell Biol..

[66] Roberts RF, Wade-Martins R, Alegre-Abarrategui J (2015). Direct visualization of alpha-synuclein oligomers reveals previously undetected pathology in Parkinson’s disease brain. Brain.

[67] Caughey B, Lansbury PT (2003). PROTOFIBRILS, PORES, FIBRILS, AND N EURODEGENERATION: separating the responsible protein aggregates from the innocent bystanders. Annu. Rev. Neurosci..

[68] Bigi A, Cascella R, Cecchi C (2023). α-Synuclein oligomers and fibrils: partners in crime in synucleinopathies. Neural. Regen. Res.

[69] Kovacs GG (2012). An antibody with high reactivity for disease-associated α-synuclein reveals extensive brain pathology. Acta. Neuropathol..

[70] Fricker M, Runions J, Moore I (2006). QUANTITATIVE FLUORESCENCE MICROSCOPY: from art to science. Annu. Rev. Plant Biol..

[71] Zinchuk V, Grossenbacher-Zinchuk O (2009). Recent advances in quantitative colocalization analysis: focus on neuroscience. Prog. Histochem. Cytochem..

[72] Eliceiri KW (2012). Biological imaging software tools. Nat. Methods.

[73] Kuusisto E, Parkkinen L, Alafuzoff I (2003). Morphogenesis of lewy bodies: dissimilar incorporation of α-synuclein, ubiquitin, and p62. J. Neuropathol. Exp. Neurol..

[74] Cappelletti G, Calogero AM, Rolando C (2021). Microtubule acetylation: a reading key to neural physiology and degeneration. Neurosci. Lett..

[75] Patel VP, Chu CT (2014). Decreased SIRT2 activity leads to altered microtubule dynamics in oxidatively-stressed neuronal cells: Implications for Parkinson’s disease. Exp. Neurol..

[76] Abeliovich A, Gitler AD (2016). Defects in trafficking bridge Parkinson’s disease pathology and genetics. Nature.

[77] Ren Y, Jiang H, Yang F, Nakaso K, Feng J (2009). Parkin protects dopaminergic neurons against microtubule-depolymerizing toxins by attenuating microtubule-associated protein kinase activation. J. Biol. Chem..

[78] Ren Y (2015). Parkin mutations reduce the complexity of neuronal processes in iPSC-derived human neurons. Stem Cells.

[79] Cartelli D (2018). Parkin absence accelerates microtubule aging in dopaminergic neurons. Neurobiol. Aging.

[80] Cartelli D, Goldwurm S, Casagrande F, Pezzoli G, Cappelletti G (2012). Microtubule destabilization is shared by genetic and idiopathic Parkinson’s disease patient fibroblasts. PLoS One.

[81] Godena VK (2014). Increasing microtubule acetylation rescues axonal transport and locomotor deficits caused by LRRK2 Roc-COR domain mutations. Nat. Commun..

[82] Fusco G (2017). Structural basis of membrane disruption and cellular toxicity by α-synuclein oligomers. Science (1979).

[83] Basso M (2004). Proteome analysis of human substantia nigra in Parkinson’s disease. Proteomics.

[84] Werner CJ (2008). Heyny-von Haussen, R., Mall, G. & Wolf, S. Proteome analysis of human substantia nigra in Parkinson’s disease. Proteom. Sci..

[85] Kitsou E (2008). Identification of proteins in human substantia nigra. Proteom. Clin. Appl.

[86] Licker V (2014). Proteomic analysis of human substantia nigra identifies novel candidates involved in Parkinson’s disease pathogenesis. Proteomics.

[87] Gai WP (2000). In situ and in vitro study of colocalization and segregation of α-synuclein, ubiquitin, and lipids in lewy bodies. Exp. Neurol..

[88] Araki K (2015). Synchrotron FTIR micro-spectroscopy for structural analysis of Lewy bodies in the brain of Parkinson’s disease patients. Sci. Rep..

[89] Even, A. et al. ATAT1-enriched vesicles promote microtubule acetylation via axonal transport. *Sci. Adv.* **5**, e0041 (2019). 10.1126/sciadv.aax2705 PMC6920029 31897425

[90] Coombes C (2016). Mechanism of microtubule lumen entry for the α-tubulin acetyltransferase enzyme αTAT1. Proc. Natl Acad. Sci..

[91] Hubbert C (2002). HDAC6 is a microtubule-associated deacetylase. Nature.

[92] North BJ, Marshall BL, Borra MT, Denu JM, Verdin E (2003). The human Sir2 Ortholog, SIRT2, is an NAD+-dependent tubulin deacetylase. Mol. Cell.

[93] Olanow CW, Perl DP, DeMartino GN, McNaught KSP (2004). Lewy-body formation is an aggresome-related process: a hypothesis. Lancet Neurol..

[94] Pandey UB (2007). HDAC6 rescues neurodegeneration and provides an essential link between autophagy and the UPS. Nature.

[95] Francelle L, Outeiro TF, Rappold GA (2020). Inhibition of HDAC6 activity protects dopaminergic neurons from alpha-synuclein toxicity. Sci. Rep..

[96] Outeiro TF (2007). Sirtuin 2 inhibitors rescue α-synuclein-mediated toxicity in models of Parkinson’s disease. Science.

[97] Esteves AR, Gozes I, Cardoso SM (2014). The rescue of microtubule-dependent traffic recovers mitochondrial function in Parkinson’s disease. Biochim. et. Biophys. Acta. BBA Mol. Basis Dis..

[98] Calogero AM (2023). Acetylated α-Tubulin and α-Synuclein: physiological interplay and contribution to α-synuclein oligomerization. Int J. Mol. Sci..

[99] Senol AD (2021). Synuclein fibrils subvert lysosome structure and function for the propagation of protein misfolding between cells through tunneling nanotubes. PLoS Biol..

[100] Valdinocci D, Kovarova J, Neuzil J, Pountney DL (2021). Alpha-synuclein aggregates associated with mitochondria in tunnelling nanotubes. Neurotox. Res..

[101] d’Ydewalle C (2011). HDAC6 inhibitors reverse axonal loss in a mouse model of mutant HSPB1–induced Charcot-Marie-Tooth disease. Nat. Med..

[102] Kim, J.-Y. et al. HDAC6 inhibitors rescued the defective axonal mitochondrial movement in motor neurons derived from the induced pluripotent stem cells of peripheral neuropathy patients with *HSPB1* mutation. *Stem Cells Int.* **2016**, 1–14 (2016). 10.1155/2016/9475981 PMC5220520 28105056

[103] Gal J (2013). HDAC6 regulates mutant SOD1 aggregation through two SMIR Motifs and tubulin acetylation. J. Biol. Chem..

[104] Lee J-Y (2015). Uncoupling of protein aggregation and neurodegeneration in a mouse amyotrophic lateral sclerosis model. Neurodegener. Dis..

[105] Dompierre JP (2007). Histone deacetylase 6 inhibition compensates for the transport deficit in Huntington’s disease by increasing tubulin acetylation. J. Neurosci..

[106] Tsushima H (2015). HDAC6 and RhoA are novel players in Abeta-driven disruption of neuronal polarity. Nat. Commun..

[107] Zhang F (2015). Posttranslational modifications of α-tubulin in Alzheimer disease. Transl. Neurodegener..

[108] Martínez-Hernández, J. et al. Crosstalk between acetylation and the tyrosination/detyrosination cycle of α-tubulin in Alzheimer’s disease. *Front. Cell Dev. Biol*. **10**, 223–251 (2022). 10.3389/fcell.2022.926914 PMC9459041 36092705

[109] Hughes AJ, Daniel SE, Kilford L, Lees AJ (1992). Accuracy of clinical diagnosis of idiopathic Parkinson’s disease: a clinico-pathological study of 100 cases. J. Neurol. Neurosurg. Psychiatr..

[110] Hughes AJ, Daniel SE, Lees AJ (2001). Improved accuracy of clinical diagnosis of Lewy body Parkinson’s disease. Neurology.

[111] Alafuzoff I (2009). Staging/typing of lewy body related α-synuclein pathology: a study of the BrainNet Europe consortium. Acta. Neuropathol..

[112] Hoehn MM, Yahr MD (1967). Parkinsonism: onset, progression, and mortality. Neurology.

[113] Filocamo M (2013). Telethon network of genetic biobanks: a key service for diagnosis and research on rare diseases. Orphanet. J. Rare Dis..

[114] Piperno G, Fuller MT (1985). Monoclonal antibodies specific for an acetylated form of alpha-tubulin recognize the antigen in cilia and flagella from a variety of organisms. J. Cell Biol..

[115] Castoldi M, Popov AV (2003). Purification of brain tubulin through two cycles of polymerization–depolymerization in a high-molarity buffer. Protein Expr. Purif..

[116] Bolte S, Cordelières FP (2006). A guided tour into subcellular colocalization analysis in light microscopy. J. Microsci..

