# Supplementary material for: Linking acetylated α-Tubulin redistribution to α-Synuclein pathology in brain of Parkinson’s disease patients
Source: NPJ Parkinsons Dis. 2024 Jan 2;10:2. doi: 10.1038/s41531-023-00607-9 (PMC10761989; doi:10.1038/s41531-023-00607-9)
Supplement: Supplementary file 1 — Supplementary information [file 41531_2023_607_MOESM1_ESM.pdf]

ADDITIONAL INFORMATION

Linking acetylated α-Tubulin redistribution to α-Synuclein pathology in brain of Parkinson's disease patients

Samanta Mazzetti[1,2, #, *], Federica Giampietro[1, #], Alessandra Maria Calogero[1,2, #], Huseyin Berkcan Isilgan[1], Gloria Gagliardi[1], Chiara Rolando[1], Francesca Cantele[3], Miriam Ascagni[4], Manuela Bramerio[5], Giorgio Giaccone[6], Ioannis Ugo Isaias[7,8], Gianni Pezzoli[2,§], Graziella Cappelletti[1,9, §, *]

[1] Department of Biosciences, Università degli Studi di Milano, Milan, Italy
[2] Fondazione Grigioni per il Morbo di Parkinson, Milan, Italy
[3] Department of Chemistry, Università degli Studi di Milano, Milan, Italy
[4] Unitech NOLIMITS, Università degli Studi di Milano, 20133 Milan, Italy
[5] S. C. Divisione Oncologia Falck and S. C. Divisione Anatomia Patologica, Ospedale Niguarda Ca' Granda, Milan, Italy
[6] Unit of Neuropathology and Neurology, Fondazione IRCCS Istituto Neurologico Carlo Besta, Milan, Italy
[7] Parkinson Institute, ASST G. Pini-CTO, Milan, Milan, Italy
[8] Department of Neurology, University Hospital of Würzburg and the Julius Maximilian University of Würzburg, 97080 Würzburg, Germany
[9] Center of Excellence on Neurodegenerative Diseases, Università degli Studi di Milano, Milan, Italy

[#] co-first
[§] co-last
[*] **Correspondence:**
Graziella Cappelletti
graziella.cappelletti@unimi.it
Samanta Mazzetti
samanta.mazzetti@gmail.com

Supplementary Figures 1-7

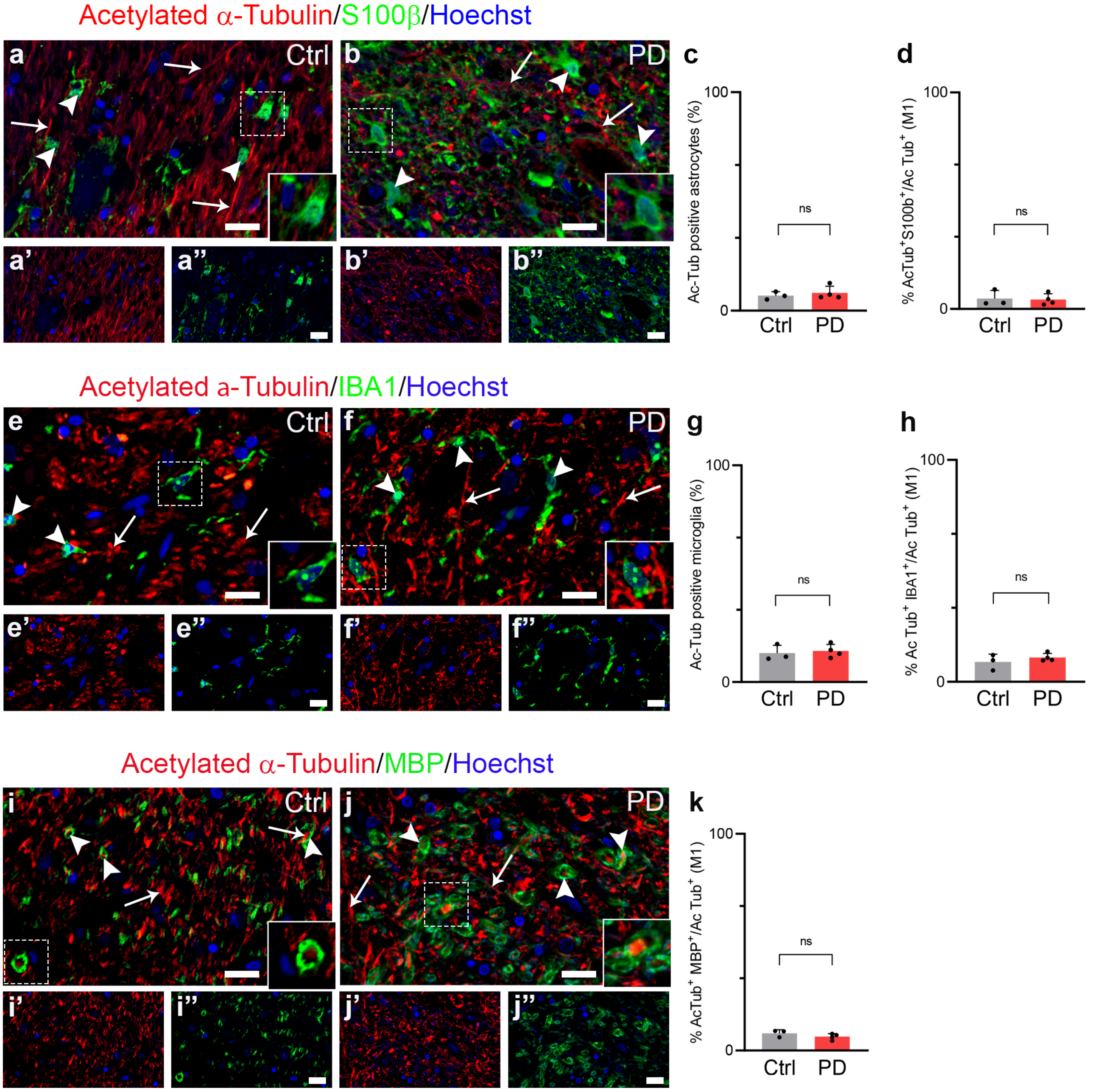

Supplementary Figure 1. Acetylated α-Tubulin (clone 6-11B-1) distribution in glial cells in *substantia nigra* of control subjects (Ctrl) and PD patients (PD). Acetylated α-Tubulin is mainly localised in neuropil (white arrows) but not in astrocyte cell bodies (S100b, **a-b''**; white arrowheads), microglia cell bodies (IBA1, **e-f'**; white arrowheads), and MBP-positive oligodendrocytes (i-j''; white arrowheads) in both control and PD samples. Insets: 1.5x magnified. Nuclei are counterstained with Hoechst. Graphs showing the quantitative analyses performed on astrocytes (**c-d**), microglial (**g-h**) and oligodendrocytes (**k**), report the percentage of glial cells positive for acetylated α-Tubulin (**c**: Ctrl, N = 3, 128 astrocytes vs PD, N = 4, 173 astrocytes; **g**: Ctrl, N = 3, 94 microglial cells vs PD, N = 4, 161 microglial cells) and the percentage of co-localisation between acetylated α-Tubulin and glial cells, expressed by Mander's coefficient (M1) (**d, h, k**). Data in graphs are reported as mean ± standard deviation; Mann-Whitney test, ns. Scale bar, 20 µm.

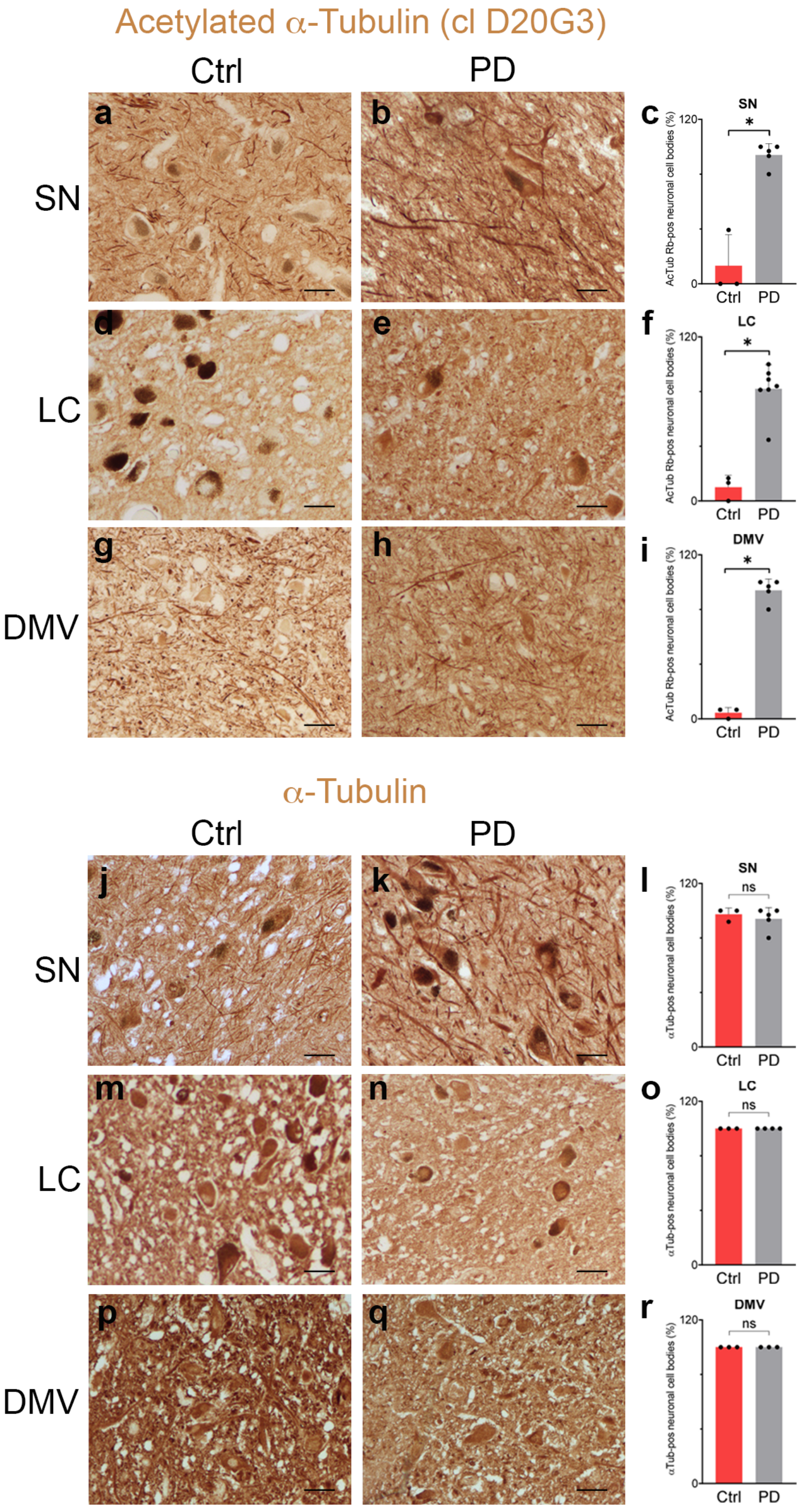

Supplementary Figure 2. Acetylated α-Tubulin (clone D20G3) and α-Tubulin distribution in *post-mortem* human brain. **a, b, d, e, g, h** Control samples show acetylated α-Tubulin only in neuropil and in axonal fibres in SN (**a**), LC (**d**) and DMV (**g**), while in PD samples (**b, e, h**), acetylated α-Tubulin is strongly localised and accumulated in neuronal cytoplasm, and is also present in neuronal processes. **j, k, m, n, p, q** total α-Tubulin is present in the neuronal cell bodies of both control (**j, m, p**) and PD samples (**k, n, q**). Scale bar 20 μm. **c, f, i, l, o, r** The graphs show the percentage of neuronal cell bodies positive for acetylated α-Tubulin (**c**: SN, Ctrl, N = 3, 149 neurons vs PD, N = 4, 235 neurons; **f**: LC, Ctrl, N = 3, 90 neurons vs PD, N = 7, 178 neurons; **i**: DMV, Ctrl, N = 3, 90 neurons vs PD, N = 5, 187 neurons) and for total α-Tubulin (**l**: SN, Ctrl, N = 3, 97 neurons vs PD, N = 5, 133 neurons; **o**: LC, Ctrl, N = 3, 90 neurons vs PD, N = 4, 60 neurons; **r**: DMV, Ctrl, N = 3, 88 neurons vs PD, N = 3, 90 neurons). Data in graphs are reported as mean ± standard deviation; Mann-Whitney test, ns, * $p < 0.05$; SN: *substantia nigra*; LC: *locus coeruleus*; DMV: dorsal motor nucleus of vagus.

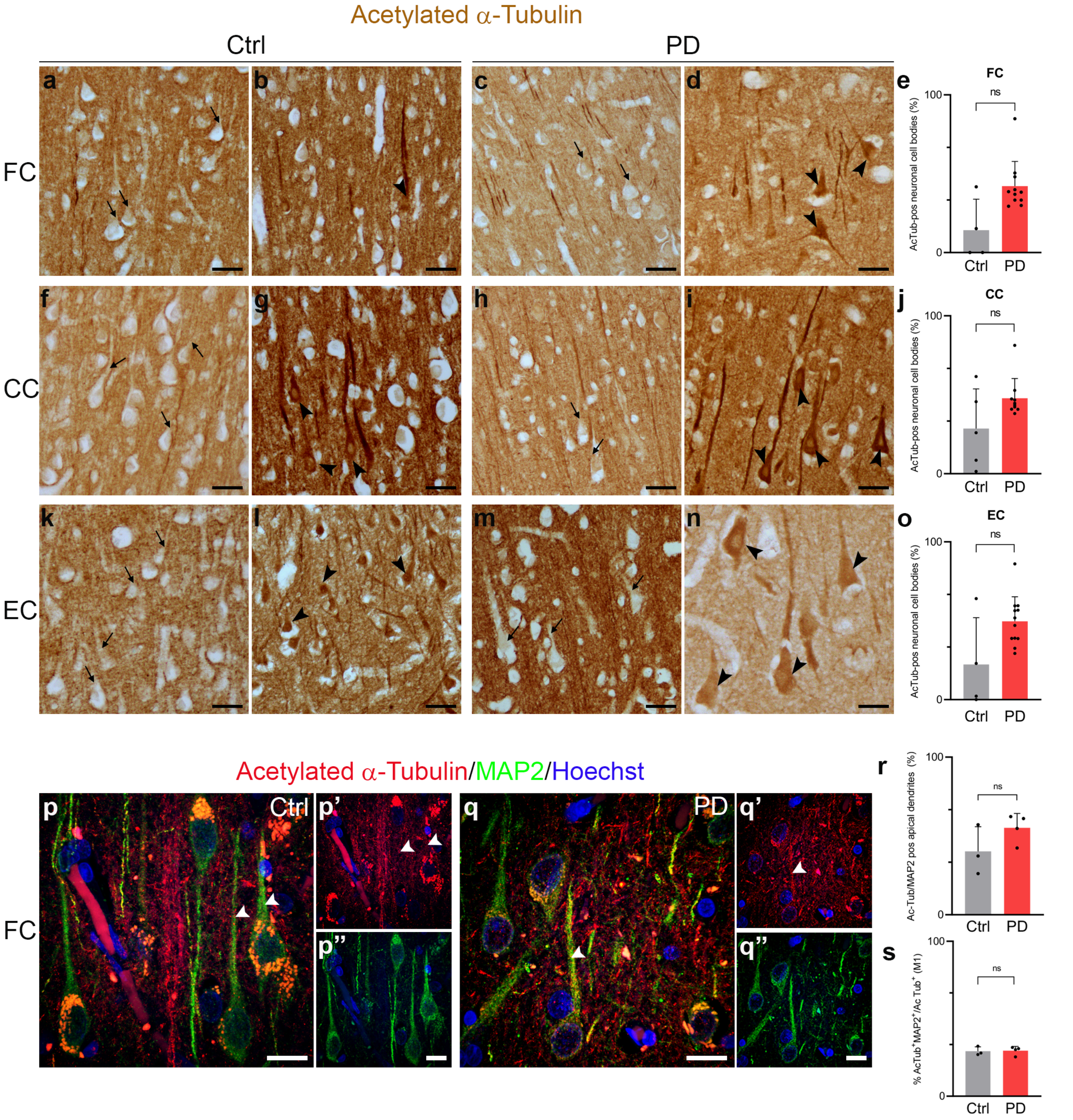

Supplementary Figure 3. Acetylated α-Tubulin (clone 6-11B-1) distribution in human cortex. **a-o** Control (**a, f, k**) and PD samples (**c, h, m**) show a homogeneous localisation of acetylated α-Tubulin inside apical dendrites (black arrows) in all three cortical regions. However, strong staining is present in the cell bodies of some pyramidal neurons (black arrowheads) in both control (**b, g, l**) and PD samples (**d, i, n**). Graphs show the percentage of acetylated α-Tubulin positive neuronal cell bodies (**e**: FC, Ctrl, N = 4, 513 neurons vs PD, N = 11, 2212 neurons; **j**: CC, Ctrl, N = 5, 672 neurons vs PD, N = 10, 1973 neurons. **o**: EC, Ctrl, N = 4, 567 neurons vs PD, N = 12, 2016 neurons.). Scale bar, 40 µm. Mann-Whitney test, ns. **p-s** MAP2 stains the pyramidal neurons both in control (**p, p''**) and PD (**q, q''**) samples. Acetylated α-Tubulin is present is some apical dendrites (white arrowheads) in controls (**p, p'**) while in PD it is also present in the soma of neurons (**q, q'**). Graphs show the percentage of apical dendrites positive for acetylated α-Tubulin (**r**; Ctrl, N = 3, 284 apical dendrites vs PD, N = 4, 428 apical dendrites) and the co-localisation between acetylated α-Tubulin and MAP2 (Mander's coefficient, M1; **s**) Nuclei are stained with Hoechst. Scale bar, 10 µm. Data in graphs are reported as mean ± standard deviation; Mann-Whitney test, ns. FC: frontal cortex; CC: cingulate cortex; EC: entorhinal cortex.

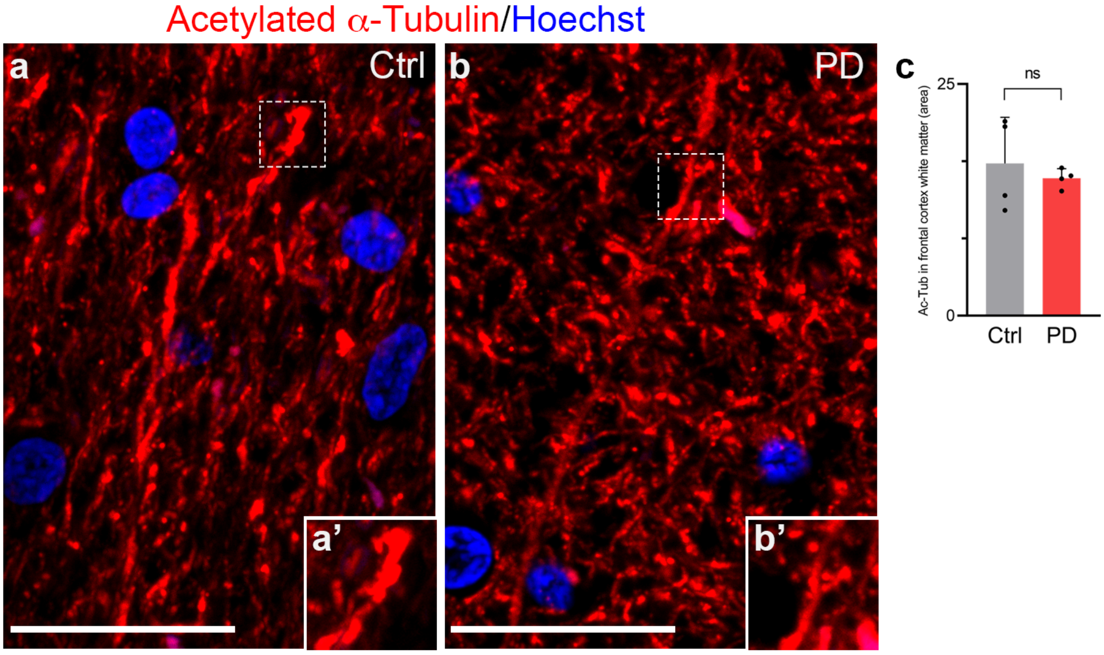

Supplementary Figure 4. Acetylated α-Tubulin (clone 6-11B-1) distribution in white matter of frontal cortex in *post-mortem* human brain. Acetylated α-Tubulin stains axonal fibres of frontal cortex white matter both in control (**a-a'**) and PD (**b-b'**) samples. Inset: 2x magnified. Nuclei are counterstained with Hoechst. Graph (**c**) showes the percentage of the area covered by acetylated α-Tubulin in fibres. Data in graphs are reported as mean ± standard deviation; Mann-Whitney test, ns. Scale bar, 20 μm.

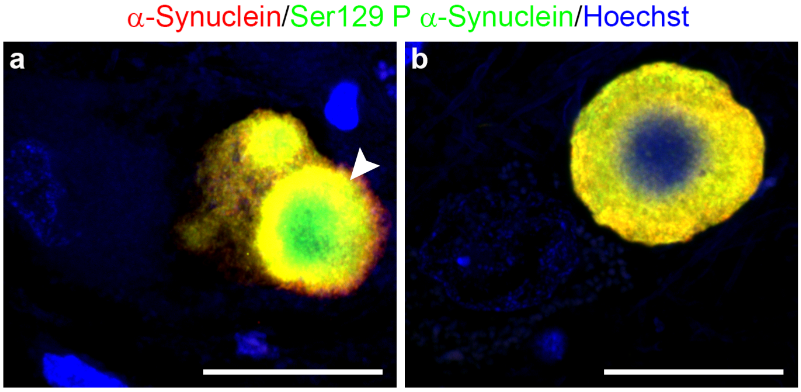

Supplementary Figure 5. α-Synuclein (S3062) and Ser129P α-Synuclein distribution in α-Synuclein aggregates of *substantia nigra* in *post-mortem* human brain of PD patients. (**a**) Total and Ser129P α-Synuclein are inside aggregates without a defined shape. Both stainings form an external ring in which they co-localise, while Ser129P α-Synuclein is also present in the core region of the structure. In a mature LB (**b**), they co-localise in an external ring and Hoechst staining is detectable inside. Nuclei are counterstained with Hoechst. Scale bar, 20 μm.

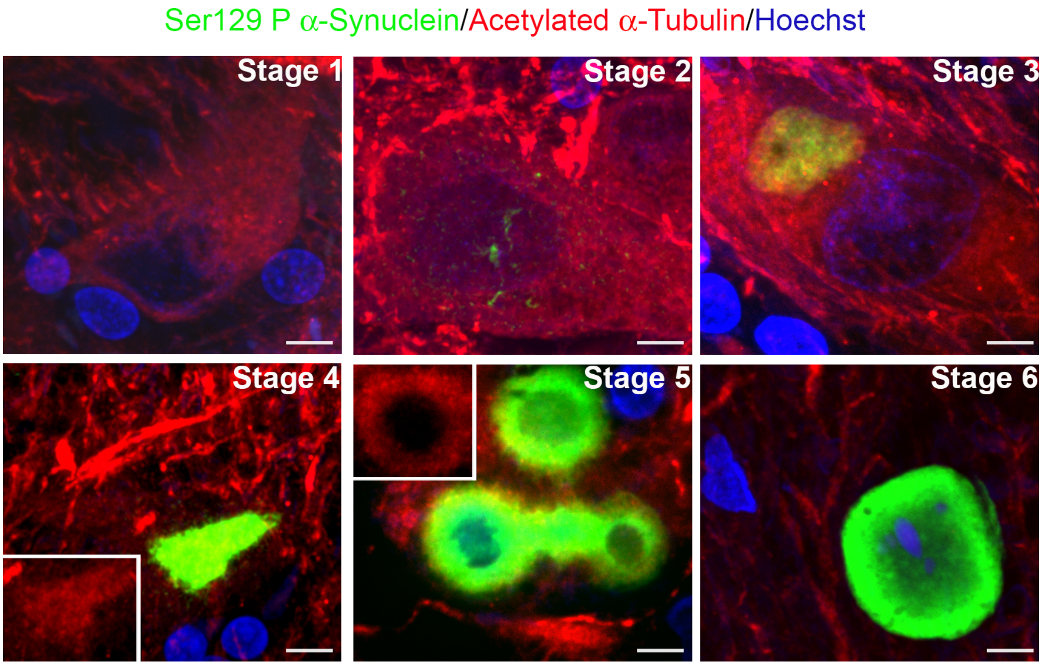

Supplementary Figure 6. Linking acetylated α-Tubulin redistribution with Phosphorylated α-Synuclein. Phosphorylated α-Synuclein (Ser129 P α-Synuclein) staining through the six different acetylated α-Tubulin (clone 6-11-B) stages distinguishable in *post-mortem* human brain sections of PD patients. Stage 1 shows the presence of acetylated α-Tubulin accumulated in the soma of neurons but no staining for phosphorylated α-Synuclein; in stage 2, acetylated α-Tubulin is accumulated inside the cell body while some phosphorylated α-Synuclein staining is detectable; in stage 3, acetylated α-Tubulin starts to accumulate into a small aggregate together with phosphorylated α-Synuclein; stage 4 shows a phosphorylated α-Synuclein aggregate that is also acetylated α-Tubulin positive; stage 5 shows a ring-shaped aggregate where acetylated α-Tubulin forms an external ring while phosphorylated α-Synuclein is distributed mainly in the external ring, but also inside it; in stage 6, the phosphorylated α-Synuclein is strongly positive in in the external part of the aggregate, whereas acetylated α-Tubulin is almost absent. Nuclei are counterstained with Hoechst. Scale bar, 20 μm.

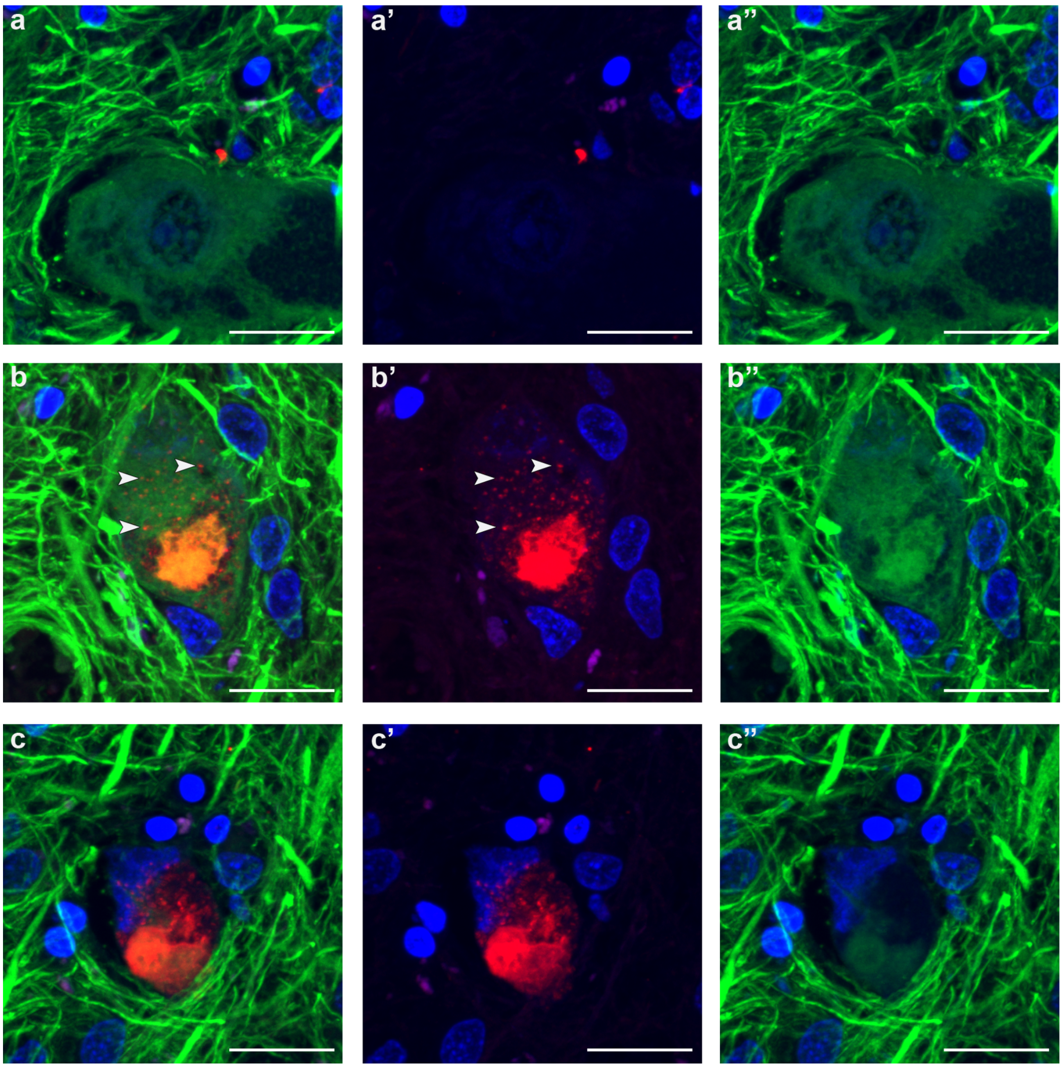

Supplementary Figure 7. Acetylated α-Tubulin (clone D20G3) and aggregated α-Synuclein (clone 5G4) distribution in α-Synuclein aggregates in *substantia nigra* of PD patients. In the early stage (a-a'', Stage 1), aggregated α-Synuclein is absent (a, a'), while the cell body is positive for acetylated α-Tubulin (a, a''). In the intermediate stage (b-b'', Stage 3), aggregated α-Synuclein is detectable in the cell body, both as little single spots (arrowheads in b, b') and in the aggregate positive for acetylated α-Tubulin (b, b''). At later stage (c-c'', Stage 5), aggregated α-Synuclein is strongly accumulated (c, c''), while acetylated α-Tubulin is not yet detectable in the cell body except for a faint staining in the external ring (c, c''). Scale bar, 20 μm.

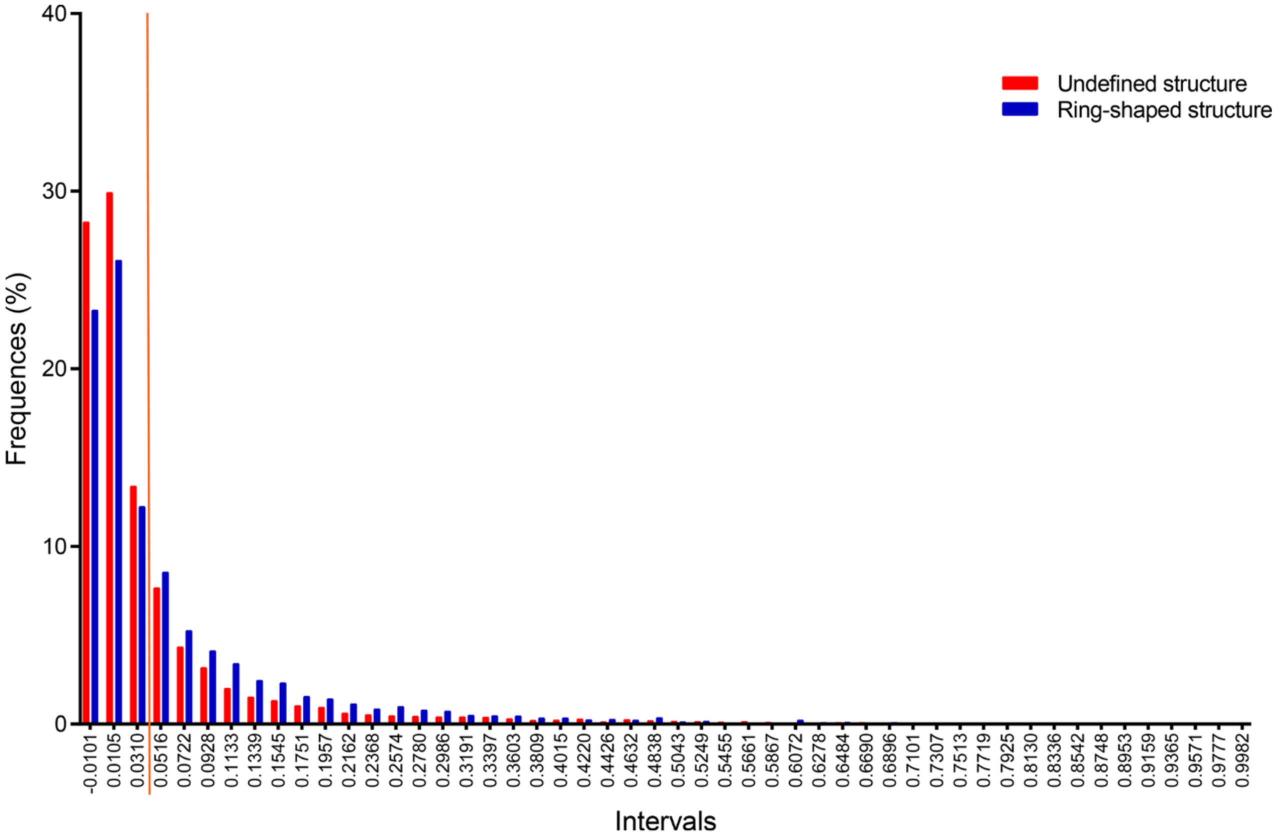

Supplementary Figure 8. Histogram of the frequencies of PLA puncta volume in undefined (red) and ring-shaped (blue) aggregates. At lower volume intervals, the frequency is higher for undefined aggregates compared to ring-shaped aggregates. The opposite is observed for PLA puncta larger than 0.0516 mm^3. The orange line distinguishes the two intervals.

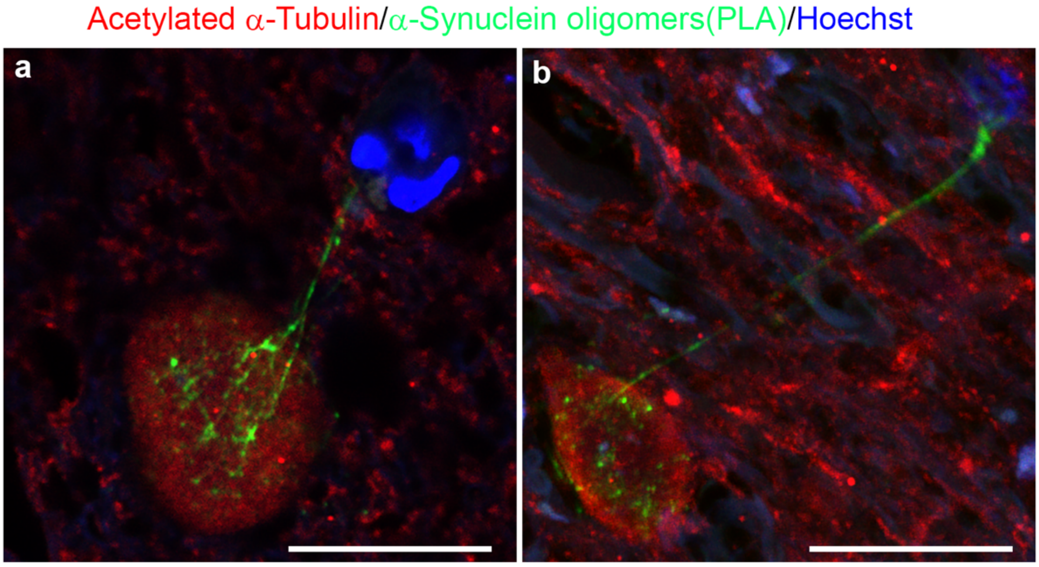

Supplementary Figure 9. Tunnelling nanotubes in *substantia nigra* of PD patients. α-Synuclein oligomers locate into threadlike structures that link a neuron accumulating acetylated α-Tubulin aggregates inside the soma to a vessel (**a**) and a glial cell (**b**). Scale bar, 25 μm.

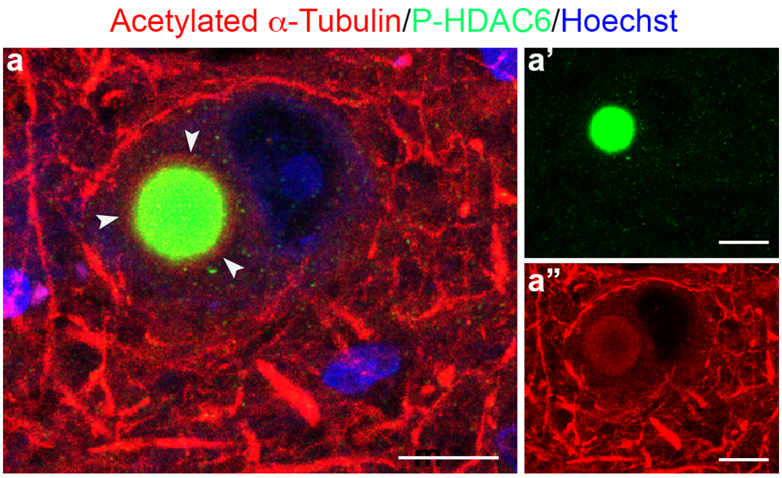

Supplementary Figure 10. Acetylated α-Tubulin (clone 6-11B-1) and Ser22 P-HDAC6 in ring shaped aggregate visualized in *substantia nigra* of PD patients. **a** Merge image showing the active form of HDAC6 deacetylase (P-HDAC6) in the acetylated α-Tubulin positive aggregate inside the neuronal cell body. P-HDAC6 is present mainly in the internal core of the structure, and a few scattered dots are visible in the outer ring (**a'**), where acetylated tubulin (**a''**) is more abundant. Arrowheads indicate the external ring only acetylated α-Tubulin positive. Nuclei are counterstained with Hoechst. Scale bar 20 μm.

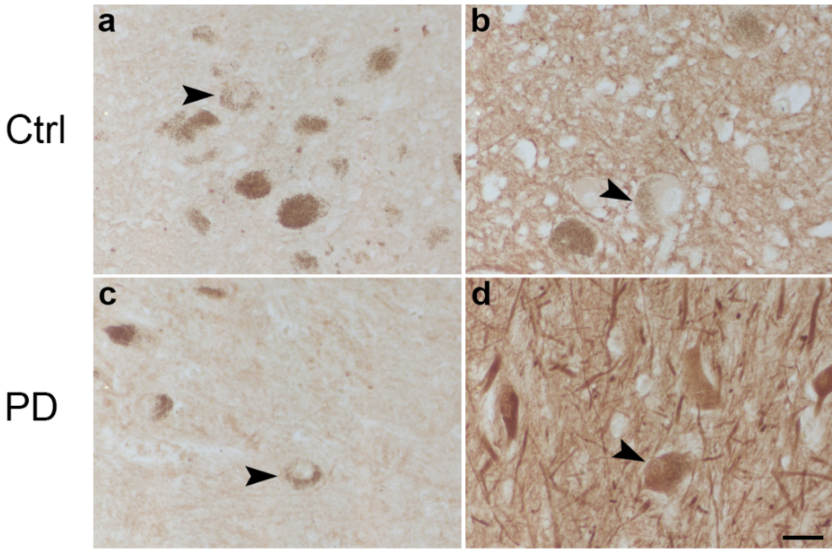

Supplementary Figure 11. Check for the specificity of acetylated α-Tubulin antibody (clone 6-11B-1) in *post-mortem* human brain. (**a-d**): Sections containing *substantia nigra* of control subjects (Ctrl) and patients affected with Parkinson's disease (PD) were incubated with anti-acetylated α-Tubulin pre-absorbed with tubulin purified from bovine brain and containing acetylated α-Tubulin (a, c), or with not-pre-absorbed anti-acetylated α-Tubulin (b, d). Staining for acetylated α-Tubulin is not detectable in sections incubated with the pre-adsorbed antibody. Dark brown signal is neuromelanin (black arrowheads). Scale bar, 40 μm.

Supplementary Tables 1-3

Subject	Gender	Age at onset	Age at death	Disease duration (year)	Hoehn and Yahr
CTRL#1	M	/	71	/	/
CTRL#2	F	/	93	/	/
CTRL#3	F	/	82	/	/
CTRL#4	F	/	64	/	/
CTRL#5	F	/	91	/	/
PD#1	M	40	59	19	5
PD#2	M	57	71	14	5
PD#3	M	57	75	18	3
PD#4	M	59	75	16	3
PD#5	M	59	87	28	3
PD#6	M	62	73	11	3
PD#7	M	62	80	18	4
PD#8	M	72	84	12	4
PD#9	F	43	72	29	5
PD#10	F	53	91	38	5
PD#11	F	59	79	20	4
PD#12	F	65	84	19	-

Supplementary Table 1. Demographic and clinical characteristics of the subjects included in the present study.

Subject	Gender	Age	Disease duration (year)
CTRL#1	M	82	/
CTRL#2	M	40	/
CTRL#3	M	66	/
CTRL#4	F	58	/
CTRL#5	M	62	/
CTRL#6	F	61	/
CTRL#7	F	57	/
CTRL#8	F	61	/
CTRL#9	M	39	/
PD#1	M	41	6
PD#2	M	56	8
PD#3	M	61	9
PD#4	F	31	7
PD#5	M	48	8
PD#6	F	69	24
PD#7	F	51	14
PD#8	M	54	1
PD#9	M	52	21
PD#10	M	71	1
PD#11	M	64	7
PD#12	F	78	9

Supplementary Table 2. Demographic and clinical characteristics of the skin biopsies included in the present study.

Primary antibodies			
Antigen	Code (clone)	Host	Dilution
α-Synuclein	S3062 Sigma-Aldrich	Rabbit	1:2000
Ser129 P α-Synuclein	ab51253 (clone EP1536Y) Abcam	Rabbit	1:1000
Aggregated α-Synuclein 5G4	MABN389 (clone 5G4) Merck Millipore	Mouse	1:1000
Acetylated α-Tubulin	T6793 (clone 6-11B-1) Sigma-Aldrich	Mouse	1:1000 (IHC) 1:500 (brain IF)/1:4000 (skin IF)
Acetylated α-Tubulin	#5335 (clone D20G3) Cell Signaling Technology	Rabbit	1:600 (IHC) 1:300 (brain IF)
α-Tubulin	T6074 (clone B-5-1-2) Sigma-Aldrich	Mouse	1:500 (IHC) 1:250 (brain IF)
Ionized calcium binding adapter molecule 1 (Iba1)	GTX 100042 GeneTex	Rabbit	1:500
Ser 22 P histone deacetylase 6 (P-HDAC6)	GTX 55403 GeneTex	Rabbit	1:50
Microtubule Associated Protein 2 (MAP2)	Ab5392 Abcam	Chicken	1:500
Myelin Basic Protein (MBP)	A0623 Dako	Rabbit	1:1000
Protein gene Product 9.5 (PGP 9.5)	AB1761-I Sigma-Aldrich	Rabbit	1:100
S100β	287006 Synaptic Systems	Chicken	1:500
Synaptophysin	GA660.61-2 (clone DAK-SYNAP) Dako Agilent	Mouse	1:100
Tau	314012 Synaptic Systems	Rabbit	1:200
Tyrosine Hydroxylase (TH)	PA-18372 Thermo Fisher	Goat	1:200
Secondary antibodies			
Fluorochrome/Enzyme Antibody	Code	Host	Dilution
Alexa Fluor® 488 anti-goat	Jackson ImmunoResearch	Donkey	1:600
Alexa Fluor® 488 anti-mouse	AB150101 Abcam	Donkey	1:200
Alexa Fluor® 568 anti-mouse	A10037 Thermo Fisher	Donkey	1:200
Alexa Fluor® 488 anti-rabbit	A21206 Thermo Fisher	Donkey	1:200
Alexa Fluor® 647 anti-rabbit	A32795 Thermo Fisher	Donkey	1:200
Cy3 anti-chicken	Jackson ImmunoResearch	Donkey	1:600
EnVision System-HRP Labelled polymer anti-mouse	K4001 Dako Agilent	Goat	1:1
EnVision System-HRP Labelled polymer anti-rabbit	K4003 Dako Agilent	Goat	1:1
ImmPRESS™-AP anti- rabbit	MP-5401Vector	Horse	1:1
Commercial assay			
Duolink® in situ probe marker MINUS	DUO920101KT Merck	-	*
Duolink® in situ probe marker PLUS	DUO920091KT Merck	-	*
Duolink® In Situ Detection Reagents Red	DUO92008 Merck	-	*
EnVision FLEX DAB+SubstrateChromogen System	K3468 Dako	-	*
Fast Blue B salt	F3378-1G Merck	-	1 mg/ml
Hoechst 33342	62249 Thermo Fisher	-	1:5000

Supplementary Table 3. Primary, secondary antibodies and kits used in this study.
* used as indicated by the manufacture instruction.

Supplementary Movies

Supplementary Movie 1. The movie refers to arivis 4D software 3D reconstruction of figure 6a'-a'''.
Supplementary Movie 2. The movie refers to arivis 4D software 3D reconstruction of figure 6b'-b'''.
Supplementary Movie 3. The movie refers to arivis 4D software 3D reconstruction of figure 6c'-c'''.
Supplementary Movie 4. The movie refers to arivis 4D software 3D reconstruction of figure 6d'-d'''.
