# Supplementary material for: Linking acetylated α-Tubulin redistribution to α-Synuclein pathology in brain of Parkinson’s disease patients
Source: NPJ Parkinsons Dis. 2024 Jan 2;10:2. doi: 10.1038/s41531-023-00607-9 (PMC10761989; doi:10.1038/s41531-023-00607-9)
Supplement: Supplementary file 2 — Reporting Summary [file 41531_2023_607_MOESM2_ESM.pdf]

nature portfolio

Corresponding author(s): Graziella Cappelletti
Samanta Mazzetti

Last updated by author(s): Oct 20, 2023

Reporting Summary

Nature Portfolio wishes to improve the reproducibility of the work that we publish. This form provides structure for consistency and transparency in reporting. For further information on Nature Portfolio policies, see our Editorial Policies and the Editorial Policy Checklist.

Statistics

For all statistical analyses, confirm that the following items are present in the figure legend, table legend, main text, or Methods section.

n/a	Confirmed	
☐	☒	The exact sample size (*n*) for each experimental group/condition, given as a discrete number and unit of measurement
☐	☒	A statement on whether measurements were taken from distinct samples or whether the same sample was measured repeatedly
☐	☒	The statistical test(s) used AND whether they are one- or two-sided<br>*Only common tests should be described solely by name; describe more complex techniques in the Methods section.*
☒	☐	A description of all covariates tested
☐	☒	A description of any assumptions or corrections, such as tests of normality and adjustment for multiple comparisons
☐	☒	A full description of the statistical parameters including central tendency (e.g. means) or other basic estimates (e.g. regression coefficient) AND variation (e.g. standard deviation) or associated estimates of uncertainty (e.g. confidence intervals)
☐	☒	For null hypothesis testing, the test statistic (e.g. *F*, *t*, *r*) with confidence intervals, effect sizes, degrees of freedom and *P* value noted<br>*Give P values as exact values whenever suitable.*
☒	☐	For Bayesian analysis, information on the choice of priors and Markov chain Monte Carlo settings
☒	☐	For hierarchical and complex designs, identification of the appropriate level for tests and full reporting of outcomes
☒	☐	Estimates of effect sizes (e.g. Cohen's *d*, Pearson's *r*), indicating how they were calculated

Our web collection on statistics for biologists contains articles on many of the points above.

Software and code

Policy information about availability of computer code

Data collection	Images were collected by a Nikon spinning disk confocal as nd2 files, converted to pseudocoloured images for visualization using Fiji.
Data analysis	Images were analysed with arivis Vision4D® software (Zeiss Company). Statistical analysis was carried out using GraphPad Prism 8.

For manuscripts utilizing custom algorithms or software that are central to the research but not yet described in published literature, software must be made available to editors and reviewers. We strongly encourage code deposition in a community repository (e.g. GitHub). See the Nature Portfolio guidelines for submitting code & software for further information.

Data

Policy information about availability of data

All manuscripts must include a data availability statement. This statement should provide the following information, where applicable:

- Accession codes, unique identifiers, or web links for publicly available datasets
- A description of any restrictions on data availability
- For clinical datasets or third party data, please ensure that the statement adheres to our policy

The datasets generated and/or analyzed during the current study are available from the corresponding authors on request.

Research involving human participants, their data, or biological material

Policy information about studies with human participants or human data. See also policy information about sex, gender (identity/presentation), and sexual orientation and race, ethnicity and racism.

Reporting on sex and gender	Gender was reported in Supplementary Tables 1 and Supplementary Table 2.
Reporting on race, ethnicity, or other socially relevant groupings	None
Population characteristics	Controls and Parkinson's disease patients. Relevant demographic and clinical data were reported in Supplementary Table 1 and Supplementary Table 2
Recruitment	All the patients were enrolled in the study and followed during the course of their disease by neurologists experienced in movement disorders and dementia at the ASST G. Pini-CTO Parkinson's Centre in Milan. Written informed consent was obtained from all subjects prior to enrolment. Post-mortem human brains were collected by the Nervous Tissues Bank (Milan, Italy). Skin biopsies were collected by the Parkinson Institute Biobank (Milan, Italy).
Ethics oversight	The study was conducted according to the guidelines of the Declaration of Helsinki and approved by the Ethics Committee of the University of Milan (protocol code 66/21, 15 June 2021).

Note that full information on the approval of the study protocol must also be provided in the manuscript.

Field-specific reporting

Please select the one below that is the best fit for your research. If you are not sure, read the appropriate sections before making your selection.

☒ Life sciences ☐ Behavioural & social sciences ☐ Ecological, evolutionary & environmental sciences

For a reference copy of the document with all sections, see nature.com/documents/nr-reporting-summary-flat.pdf

Life sciences study design

All studies must disclose on these points even when the disclosure is negative.

Sample size	The sample size of each experiment is provided in the corresponding main figure legend, main text or Material and methods section.
Data exclusions	No data were excluded from analysis.
Replication	Measurements were conducted as specified in the text and/or figure legends.
Randomization	None
Blinding	Procedures to evaluate cells positive for alpha Tubulin or acetylated Tubulin were performed blind to the disease .

Reporting for specific materials, systems and methods

We require information from authors about some types of materials, experimental systems and methods used in many studies. Here, indicate whether each material, system or method listed is relevant to your study. If you are not sure if a list item applies to your research, read the appropriate section before selecting a response.

Materials & experimental systems

n/a	Involved in the study	
☐	☒	Antibodies
☒	☐	Eukaryotic cell lines
☒	☐	Palaeontology and archaeology
☒	☐	Animals and other organisms
☒	☐	Clinical data
☒	☐	Dual use research of concern
☒	☐	Plants

Methods

n/a	Involved in the study	
☒	☐	ChIP-seq
☒	☐	Flow cytometry
☒	☐	MRI-based neuroimaging

Antibodies

Antibodies used	All the antibodies used are commercial and listed in Supplementary Table 3
Validation	α-Synuclein Product No: S3062; Sigma-Aldrich; host:Rabbit; Reactivity: human [validated by Calogero, A. M. et al. Acetylated α-Tubulin and α-Synuclein: Physiological Interplay and Contribution to α-Synuclein Oligomerization. Int J Mol Sci 24, 12287 (2023)].<br><br>Ser129P-α-Synuclein (clone EP1536Y) Product No: ab51253; Abcam; host:Rabbit; Reactivity: human [see for validation Delic V. et al. Sensitivity and specificity of phospho-Ser129 α-synuclein monoclonal antibodies. J Comp Neurol. 526(12):1978-1990 (2018)].<br><br>Aggregated-α-Synuclein (clone 5G4) Product No: MABN389; Merck Millipore; host: Mouse; Reactivity: human [see for validation Kovacs, G. G. et al. An antibody with high reactivity for disease-associated α-synuclein reveals extensive brain pathology. Acta Neuropathol 124, 37–50 (2012)].<br><br>Acetylated α-Tubulin (Clone 6-11B-1); Product No: T6793; Sigma-Aldrich; host: Mouse; Reactivity: human; validate by the manufacture.<br><br>Acetylated α-Tubulin (clone D20G3); Product No: #5335; Cell Signaling Technology; host: Rabbit; Reactivity: Human; validate by the manufacture.<br><br>α-Tubulin (clone B-5-1-2); Product No: T6074; Sigma-Aldrich; host: Mouse; Reactivity: human; validate by the manufacture.<br><br>Ionized calcium binding adapter molecule 1 (Iba1); Product No: GTX 100042; GeneTex; host: Rabbit, Reactivity Human; validate by the manufacture.<br><br>Ser 22 P histone deacetylase 6 (P-HDAC6); Product No: GTX 55403; GeneTex; host: Rabbit, Reactivity: Human; Validated in [Mazzetti, S. et al. Phospho-HDAC6 Gathers Into Protein Aggregates in Parkinson's Disease and Atypical Parkinsonisms. Front Neurosci 14, (2020)].<br><br>Microtubule Associated Protein 2 (MAP2), Product No: Ab5392; Abcam; host: Chicken; Reactivity: human; validated by the manufacture.<br><br>Myelin Basic Protein (MBP) Product No: A0623; Dako; Host: Rabbit; Reactivity: human; validated by the manufacture.<br><br>Protein gene Product 9.5 (PGP 9.5), Product No AB1761-I, Host: Rabbit; Reactivity: human; validated by the manufacture;<br><br>S100 calcium binding protein B (S100β) Product No 287006; Synaptic System; host: Chicken; Reactivity: human; validated by the manufacture.<br><br>Synaptophysin (Clone DAK-SYNAP) Product No GA660.61-2; Dako Agilent; Host: Mouse; diagnostic validated; Reactivity: human.<br><br>Tau Product No 314012; Synaptic System; host: Rabbit; Reactivity: human; Recombinant protein corresponding to AA 1 to 44 from human TauE, validated by the manufacture.<br><br>Tyrosine Hydroxylase (TH) Product No PA-18372; Thermo Fisher; host: Goat; Reactivity: human; validated by the manufacture.

Plants

Seed stocks	*Report on the source of all seed stocks or other plant material used. If applicable, state the seed stock centre and catalogue number. If plant specimens were collected from the field, describe the collection location, date and sampling procedures.*
Novel plant genotypes	*Describe the methods by which all novel plant genotypes were produced. This includes those generated by transgenic approaches, gene editing, chemical/radiation-based mutagenesis and hybridization. For transgenic lines, describe the transformation method, the number of independent lines analyzed and the generation upon which experiments were performed. For gene-edited lines, describe the editor used, the endogenous sequence targeted for editing, the targeting guide RNA sequence (if applicable) and how the editor was applied.*
Authentication	*Describe any authentication procedures for each seed stock used or novel genotype generated. Describe any experiments used to assess the effect of a mutation and, where applicable, how potential secondary effects (e.g. second site T-DNA insertions, mosiacism, off-target gene editing) were examined.*
